# Application of the Heavy-Atom Effect for (Sub)microsecond Thermally Activated Delayed Fluorescence and an All-Organic Light-Emitting Device with Low-Efficiency Roll-off

**DOI:** 10.1021/acsami.3c19627

**Published:** 2024-03-18

**Authors:** Michał Mońka, Szymon Gogoc, Karol Kozakiewicz, Vladyslav Ievtukhov, Daria Grzywacz, Olga Ciupak, Aleksander Kubicki, Piotr Bojarski, Przemysław Data, Illia E. Serdiuk

**Affiliations:** † Faculty of Mathematics, Physics and Informatics, University of Gdańsk, Wita Stwosza 57, 80-308 Gdańsk, Poland; ‡ Faculty of Materials Science and Ceramics, AGH University of Krakow, Mickiewicza 30, 30-059 Krakow, Poland; § Faculty of Chemistry, University of Gdańsk, Wita Stwosza 63, 80-308 Gdańsk, Poland; ∥ Department of Organic Chemistry, Gdańsk University of Technology, Gabriela Narutowicza 11/12, 80-233 Gdańsk, Poland; ⊥ Faculty of Chemistry, Department of Molecular Physics, Lodz University of Technology, Zeromskiego 116, 90-543 Lodz, Poland

**Keywords:** TADF, OLEDs, heavy-atom effect, organic emissive materials, FRET, hyperfluorescence

## Abstract

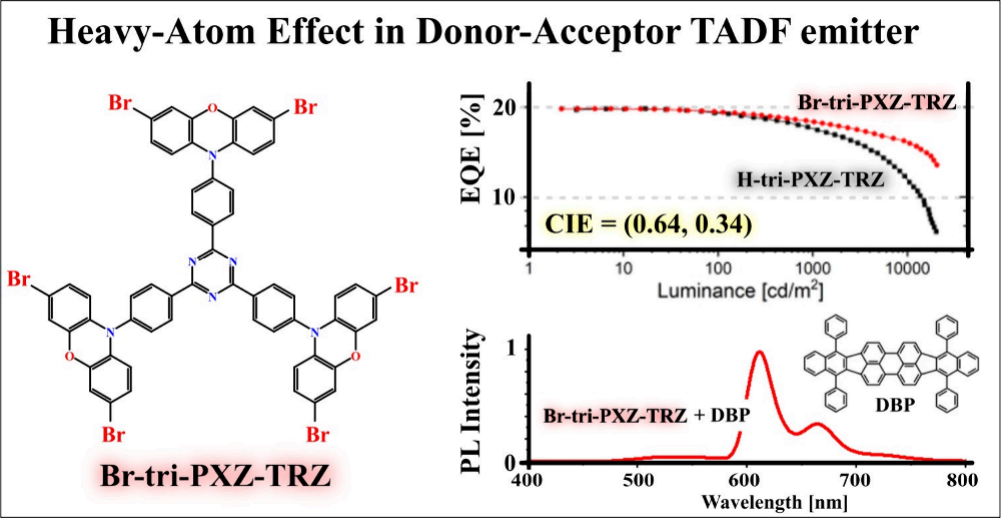

The feature of abundant and environmentally friendly heavy atoms (HAs) like bromine to accelerate spin-forbidden transitions in organic molecules has been known for years. In combination with the easiness of incorporation, bromine derivatives of organic emitters showing thermally activated delayed fluorescence (TADF) emerge as a cheap and efficient solution for the slow reverse intersystem crossing (rISC) problem in such emitters and strong efficiency roll-off of all-organic light-emitting diodes (OLEDs). Here, we present a comprehensive photophysical study of a **tri-PXZ-TRZ** emitter reported previously and its hexabromo derivative showing a remarkable enhancement of rISC of up to 9 times and a short lifetime of delayed fluorescence of 2 μs. Analysis of the key molecular vibrations and TADF mechanism indicates almost compete blockage of the spin-flip transition between the charge-transfer states of different multiplicity ^3^CT → ^1^CT. In such a case, rISC as well as its enhancement by the HA is realized via the ^3^LE → ^1^CT transition, where ^3^LE is the triplet state localized on the same brominated phenoxazine donor involved in the formation of the ^1^CT state. Interestingly, the spin–orbit coupling (SOC) with two other ^3^LE states is negligible because they are localized on different donors and not involved in ^1^CT. We consider this as an example of an additional “localization” criterion that completes the well-known El Sayed rule on the different nature of states for nonzero SOC. The applicative potential of such a hexabromo emitter is tested in a “hyperfluorescent” system containing a red fluorescent dopant (tetraphenyldibenzoperiflanthene, DBP) as an acceptor of Förster resonance energy transfer, affording a narrow-band red-emitting system, with most of the emission in the submicrosecond domain. In fact, the fabricated red OLED devices show remarkable improvement of efficiency roll-off from 2–4 times depending on the luminance, mostly because of the increase of the rISC constant rate and the decrease of the overall delayed fluorescence lifetime thanks to the HA effect.

## Introduction

Thermally activated delayed fluorescence (TADF) has emerged as a groundbreaking phenomenon in the field of heavy-metal-free organic optoelectronics^1^ because it allows one to utilize 100% excitons in organic light-emitting diodes (OLEDs). Thanks to triplet harvesting via the reverse intersystem crossing (rISC) process, TADF emitters have great potential for enhancing the stability and improving the efficiency of OLEDs, as was evidenced by numerous recent studies.

In principle, the realization of TADF in organic emitters requires minimization of the energy gap between the lowest excited triplet and singlet excited states (Δ*E*_ST_).^2^ In practice, this requirement can be met in donor–acceptor (D–A) systems with large D–A dihedral angles, resulting in charge-transfer (CT) character of excited states.^3^ However, most of the developed OLED devices based on organic TADF-active emitters still suffer from a strong efficiency roll-off and low stability as a consequence of the accumulation of long-lived triplet excitons. In fact, a spin-flip transition between the states of the same CT nature is a forbidden process, and its spin–orbit coupling (SOC) is too low to enable rISC in a submicrosecond domain to meet the application requirements. Therefore, the major problem that needs to be solved is to accelerate rISC and shorten the TADF lifetime below a few microseconds in heavy-metal-free organic emitters to ensure the efficient and stable conversion of electricity to light.

To facilitate rISC, one can use a locally excited triplet state (^3^LE) because, according to the El Sayed rules,^4^ the strength of the SOC is higher between states of different nature.^5,6^ One of the popular molecular design strategies thus involves the introduction of multiple donor and/or acceptor fragments, which should increase the density of ^3^LE excited states.^7^ As a negative factor, the energetic closeness of the ^3^LE and ^1^CT states can enhance undesired direct intersystem crossing (ISC), which populates triplet states.^8^ Therefore, from the point of view of the application in OLEDs, it is crucial to avoid fast ISC.

On the other hand, the introduction of cheap and abundant heavy atoms (HAs) seems to be a rational way to increase SOC and accelerate rISC thanks to the HA effect. Although this approach can potentially enhance ISC, our previous investigations showed that the selective acceleration of rISC can be achieved by a controlled incorporation of bromine atoms into the structure of TADF emitters.^9^ Analysis of the literature also evidenced that the various modifications of TADF emitters with HAs can significantly improve their photophysical kinetic properties.^10−16^

**Scheme 1:**
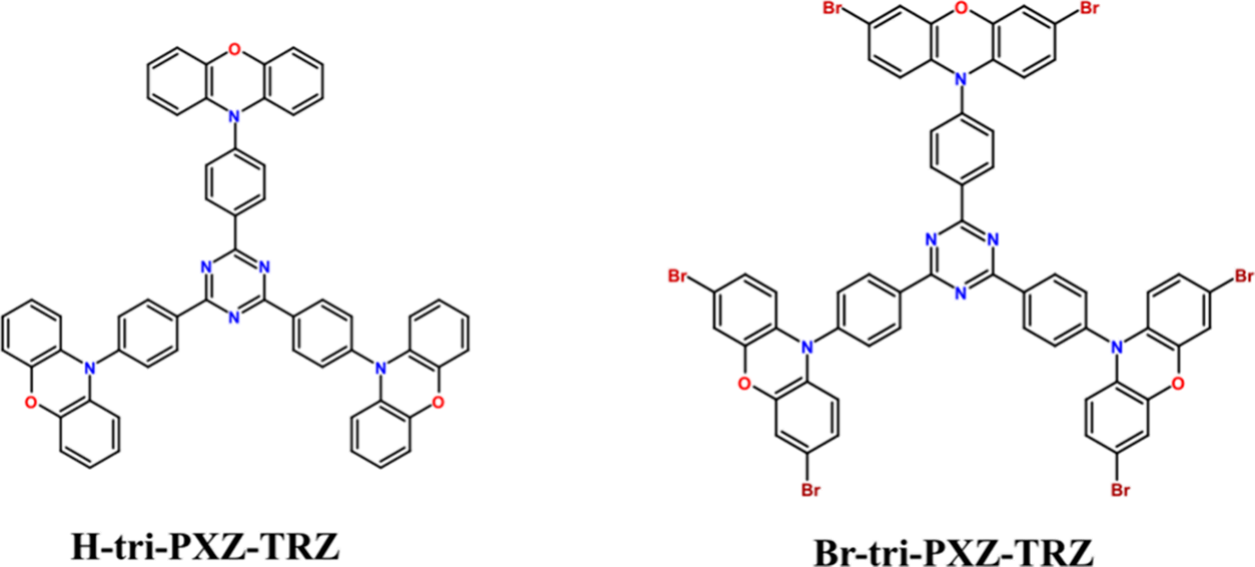
Structures of the Investigated Emitters

In this paper, we combine both of these approaches and present a comprehensive study of the photophysical properties of a previously reported tridonor–acceptor TADF emitter^17,18^ (**H-tri-PXZ-TRZ**) and its brominated derivative (**Br-tri-PXZ-TRZ**), as depicted on Scheme 1. Experimental results supported by theoretical calculations reveal a strong HA effect on the rISC rate via the ^3^LE–^1^CT transition in **Br-tri-PXZ-TRZ** but a negligible effect of the multiplication of donor fragments. The fabricated red “hyperfluorescent” OLEDs comprising **Br-tri-PXZ-TRZ** showed considerable suppression of the efficiency roll-off due to the shortened TADF lifetime while maintaining the maximal external quantum efficiency (EQE_max_) at 20%.

## Materials and Methods

### Materials

Zeonex (ZNX; Zeonex480R; density = 1.01 g/cm^3^), 4,4′-bis(*N*-carbazolyl)-1,10-biphenyl (CBP), bis[2-(diphenylphosphino)phenyl]ether oxide (DPEPO), tetraphenyldibenzoperiflanthene (DBP), reagents for synthesis, and solvents of respective grades for spectroscopy were purchased and used without further purification.

### Synthesis

**H-tri-PXZ-TRZ** was synthesized as described previously.^17^**Br-tri-PXZ-TRZ** and 3,7-dibromo-10*H*-phenoxazine were obtained by bromination of **H-tri-PXZ-TRZ** and phenoxazine with an appropriate amount of brominating agent, *N*-bromosuccinimide.

### 2,4,6-Tris-4-(3,7-dibromo-10*H*-phenoxazin-10-yl)benzene-1,3,5-triazine (**Br-tri-PXZ-TRZ**)

A total of 1 mmol of **H-tri-PXZ-TRZ** and 6.6 mmol of *N*-bromosuccinimide were dissolved in 20 mL of chloroform and stirred for 12 h in the dark. The solvent was evaporated, and the residue was treated with methanol. The resulting precipitate was collected and purified by column chromatography on SiO_2_ using the appropriate hexane/chloroform mixtures as eluents. **Br-tri-PXZ-TRZ** was obtained as a yellow powder in a yield of 87%. ^1^H NMR (500 MHz, CDCl_3_, δ): 5.92 (d, *J* = 8.4 Hz, 6 H), 6.77 (dd, *J* = 8.4 and 2.2 Hz, 6 H), 6.90 (d, *J* = 2.0 Hz, 6 H), 7.59 (d, *J* = 8.6 Hz, 6 H), 9.04 (d, *J* = 8.6 Hz, 6 H). ^13^C NMR (125 MHz, CDCl_3_, δ): 171.2, 144.2, 142.5, 136.2, 132.7, 132.1, 130.9, 126.4, 118.9, 114.4, 113.4. MALDI-TOF. Calcd for C_57_H_30_N_6_O_3_Br_6_: *m*/*z* 1325.78 ([M]^+^). Found: *m*/*z* 1325.84. For NMR and MALDI-TOF spectra, see the Supporting Information (SI).

### Sample Preparation for Photophysical Measurements

Films in ZNX, CBP, and DPEPO were prepared by a solution-processing technique using a spin-coating method.

### UV–Vis and Photoluminescence (PL) Measurements

UV–vis absorption spectra were recorded using a Shimadzu UV-1900 spectrophotometer. Steady-state PL measurements were conducted using a FS5 spectrofluorometer (Edinburgh Instruments) using front-face excitation geometry with a 1 nm spectral resolution. Absolute photoluminescence quantum yields (PLQYs) were measured using an integrating sphere, included in the FS5 spectrofluorometer. Time-resolved measurements were performed using a customized system^19^ consisting of a pulsed YAG:Nd laser (PL2251A, EKSPLA) coupled with an optical parametric generator (PG 401/SH) as the excitation light source and a 2501S grating spectrometer (Bruker Optics) combined with a streak camera system (C4334-01, Hamamatsu) as the detection unit. The system was equipped with a double-stage high-vacuum pump (T-Station 85, Edwards). To reduce scattering, reflections, and secondary order artifacts, a set of various high-performance optical bandpass (BP) and long-pass (LP) filters were used, in the excitation path 325/50BP, together with a LP filter 375LP (Edmund Optics). To build PL intensity decay profiles, streak camera images were integrated over a constant specified wavelength interval. Phosphorescence measurements were recorded at 10 K using a closed-cycle helium cryostat (APD DE-202) and a temperature controller (LakeShore 336). The photophysical constant rates *k*_r_, *k*_nr_, *k*_ISC_, and *k*_rISC_ were calculated according to equations described in Section S1.

### Quantum-Chemical Calculations

Quantum-chemical calculations were conducted at the the density functional theory (DFT)/time-dependent density functional theory (TD-DFT) level of theory using the *Gaussian 16* program package.^20^ The B3LYP functional was used with the LAN2LDZ basis set.^21^ SOC constants were computed using the *ORCA 4.2* software package^22^ with the B3LYP functional and DEF2-TZVP basis set with the relativistic zero-order regular approximation included.

### Electroluminescence (EL) Measurements

The materials for OLED fabrication were purchased from Sigma-Aldrich or Lumtec and purified by temperature-gradient sublimation in a vacuum, except for the polymers. Poly(3,4-ethylenedioxythiophene):poly(styrenesulfonate) (PEDOT:PSS) Al4083 and *N*,*N*′-bis(1-naphthyl)-*N*,*N*′-diphenyl-(1,1′-biphenyl)-4,4′-diamine (NPB) were used as a hole-injection layer (HIL) and a hole-transport layer (HTL), respectively, and 2,2′,2″-(1,3,5-benzinetriyl)tris(1-phenyl-1*H*-benzimidazole) (TPBi) and 1,3,5-tris(*m*-pyridin-3-ylphenyl)benzene (TmPyPB) were introduced as electron-transport layers (ETLs). Lithium fluoride (LiF) and aluminum (Al) were used as cathodes. The solution-processed hyperfluorescence (HF)-TADF-OLEDs were fabricated using the spin-coating method with concentrations of 3–30% (w/w) of the emitters and 1–3% (w/w) of the DBP dopant in the CBP host. The device configuration was indium–tin oxide (ITO)/PEDOT:PSS-Clevios (40 nm)/emitter + CBP (30 nm)/TPBi (50 nm)/LiF (1 nm)/Al (100 nm), with the last three layers deposited by evaporation. PEDOT:PSS was spin-coated after filtering at 3000 rpm for 45 s, resulting in 40 nm layers, and annealed at 120 °C for 15 min. The emitters in chloroform/chlorobenzene (95:5, v/v) were spin-coated at 3000 rpm for 45 s, resulting in 30 nm thickness, with no annealing. After that, the obtained samples were moved to a vacuum chamber for deposition of the remaining layers. In the case of vacuum-deposited OLEDs, organic semiconductors and Al were deposited at a rate of 1 Å/s, and the LiF layer was deposited at 0.1 Å/s. CBP was used as the host for both emitters. OLEDs were fabricated on precleaned, patterned ITO-coated glass substrates with a sheet resistance of 20 Ω/sq and a ITO thickness of 100 nm. All small molecules and cathode layers were thermally evaporated in a Kurt J. Lesker Spectros evaporation system under a pressure of 10^–7^ mbar without breaking the vacuum. The sizes of the pixels were 4, 8, and 16 mm^2^. Each emitting layer was formed by multiple depositions of the TADF emitter, assistant dopant, and host at a specific rate to obtain the particular content of the materials. The characteristics of the devices were recorded using a 6-in. integrating sphere (Labsphere) inside the glovebox connected to a sourcemeter unit and an Ocean Optics USB4000 spectrometer.

## Results and Discussion

### PL Properties

**Figure 1:**
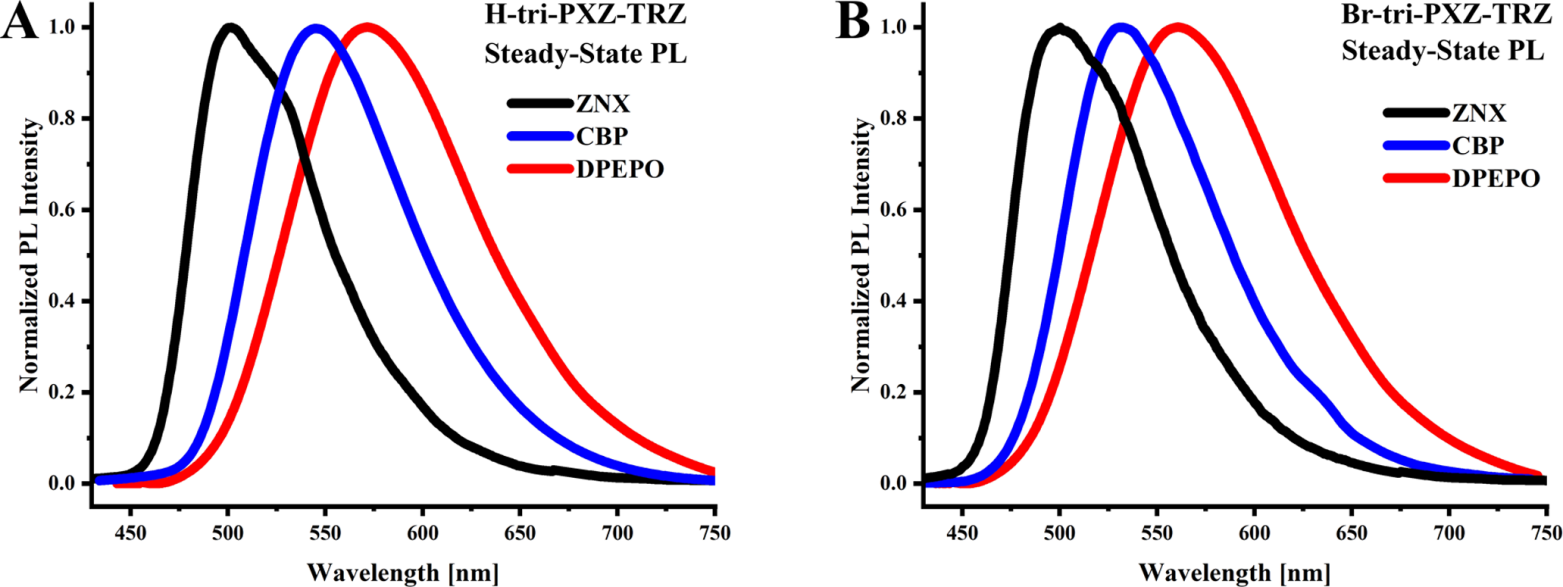
Steady-state PL spectra of **H-tri-PXZ-TRZ** (A) and **Br-tri-PXZ-TRZ** (B) measured in 0.1% ZNX, 10% CBP, and 10% DPEPO (w/w) hosts at room temperature. Excitation wavelength = 330 nm.

The photophysical investigations started with steady-state PL measurements in three different media: ZNX, CBP and DPEPO hosts. The PLQYs (Φ_PL_) are the highest in ZNX (Φ_PL_ = 94% and 90% for **H-tri-PXZ-TRZ** and **Br-tri-PXZ-TRZ**, respectively), whereas in CBP, the Φ_PL_ values decrease to 62% and 60% and further, in DPEPO, Φ_PL_ = 33% and 30%, respectively (Table 1). As can be seen in Figure 1, both emitters show positive solvatofluorochromism, typical for most of the TADF emitters, with a large change in the dipole moment during the S_1_–S_0_ transition and CT character of the S_1_ state. In nonpolar ZNX, a relatively narrow emission band is observed, with the fluorescence onsets (λ_onset_) at 462 and 459 nm, which gives ^1^CT state energies of 2.70 and 2.69 eV for **H-tri-PXZ-TRZ** and **Br-tri-PXZ-TRZ**, respectively. In the more polar CBP and DPEPO, the PL spectra become structureless and broad and shift to longer wavelengths, resulting in lower ^1^CT state energies of 2.56 and 2.59 eV in CBP and 2.50 and 2.55 eV in DPEPO, respectively.

**Table 1:** Steady-State PL Parameters of Investigated TADF Emitters

cmpd	medium	Φ_PL_^a^ [%]	PL_max_ [nm]	*E*(^1^CT)^b^/*λ*_onset_^c^ [eV]/[nm]	*E*(^3^CT)^b^/*λ*_onset_^c^ [eV]/[nm]	*E*(^3^LE)^b^/*λ*_onset_^c^ [eV]/[nm]	Δ*E*_^1^CT–^3^CT_^d^ [eV]	Δ*E*_^1^CT–^3^LE_^d^ [eV]
**H-tri-PXZ-TRZ**	ZNX	94	502	2.69/462	2.60/–	2.59/478	0.09	0.10
**Br-tri-PXZ-TRZ**		90	499	2.70/459	2.61/–	2.58/482	0.10	0.12
**H-tri-PXZ-TRZ**	CBP	62	545	2.56/484	2.49/498	2.59/–	0.07	–0.04
**Br-tri-PXZ-TRZ**		60	530	2.59/478	2.51/495	2.58/–	0.08	0.01
**H-tri-PXZ-TRZ**	DPEPO	33	575	2.50/496	2.44/507	2.59/–	0.06	–0.09
**Br-tri-PXZ-TRZ**		30	550	2.55/486	2.48/502	2.58/–	0.07	–0.03

a PLQY values corrected for vacuum conditions. The presented values were determined with ±5% error, according to equipment specifications.^23^

b The energies of the respective excited states.

c The onsets of the PL spectra.

d The energy gaps between the ^1^CT and ^3^CT or ^3^LE_D_ excited states, respectively.

The effect of the introduction of six bromine atoms to the **H-tri-PXZ-TRZ** molecule on the spectral properties in different media is shown in Figure S1. The PL spectrum of **Br-tri-PXZ-TRZ** is blue-shifted relative to that of **H-tri-PXZ-TRZ** as a result of the negative inductive effect of bromine atoms, which decreases the donor strength (**PXZ**) and thus increases the ^1^CT energy. The differences in the *λ*_onset_ values between **H-tri-PXZ-TRZ** and **Br-tri-PXZ-TRZ** are higher in more polar media because of stronger host–emitter interactions, which stabilize and differentiate the CT states.

To reveal the nature and energy of the lowest triplet excited states, low-temperature measurements were performed. Figure S2A presents the phosphorescence spectra of emitters dispersed in a ZNX film recorded at 10 K with a 10 ms delay. The shapes of the spectra of both emitters indicate the LE character of the T_1_–S_0_ transition because their vibrational structures are very similar to the phosphorescence spectra of isolated **PXZ** and **diBr-PXZ** donor fragments, respectively (Figure S3A–C). In ZNX, the phosphorescence of **Br-tri-PXZ-TRZ** is red-shifted relative to that of **H-tri-PXZ-TRZ**, and thus the T_1_ energy *E*(T_1_) is equal to 2.58 and 2.59 eV, respectively. Such a change is most likely due to the decrease of the highest occupied molecular orbital (HOMO) energy, evidencing the direct influence of bromine atoms on such an T_1_ state localized on the donor fragment (^3^LE_D_). In contrast to this, in 10% CBP and DPEPO, the phosphorescence spectra of **Br-tri-PXZ-TRZ** shown in Figure S2B,C are blue-shifted relative to those of **H-tri-PXZ-TRZ**. This, together with their broad and structureless shape, indicates strong CT character of such a T_1_–S_0_ transition, similar to that of S_1_–S_0_ observed in the PL spectra (Figure S1). Hence, in more polar CBP and DPEPO hosts, the lowest triplet excited state of both emitters is attributed to ^3^CT.

**Figure 2:**
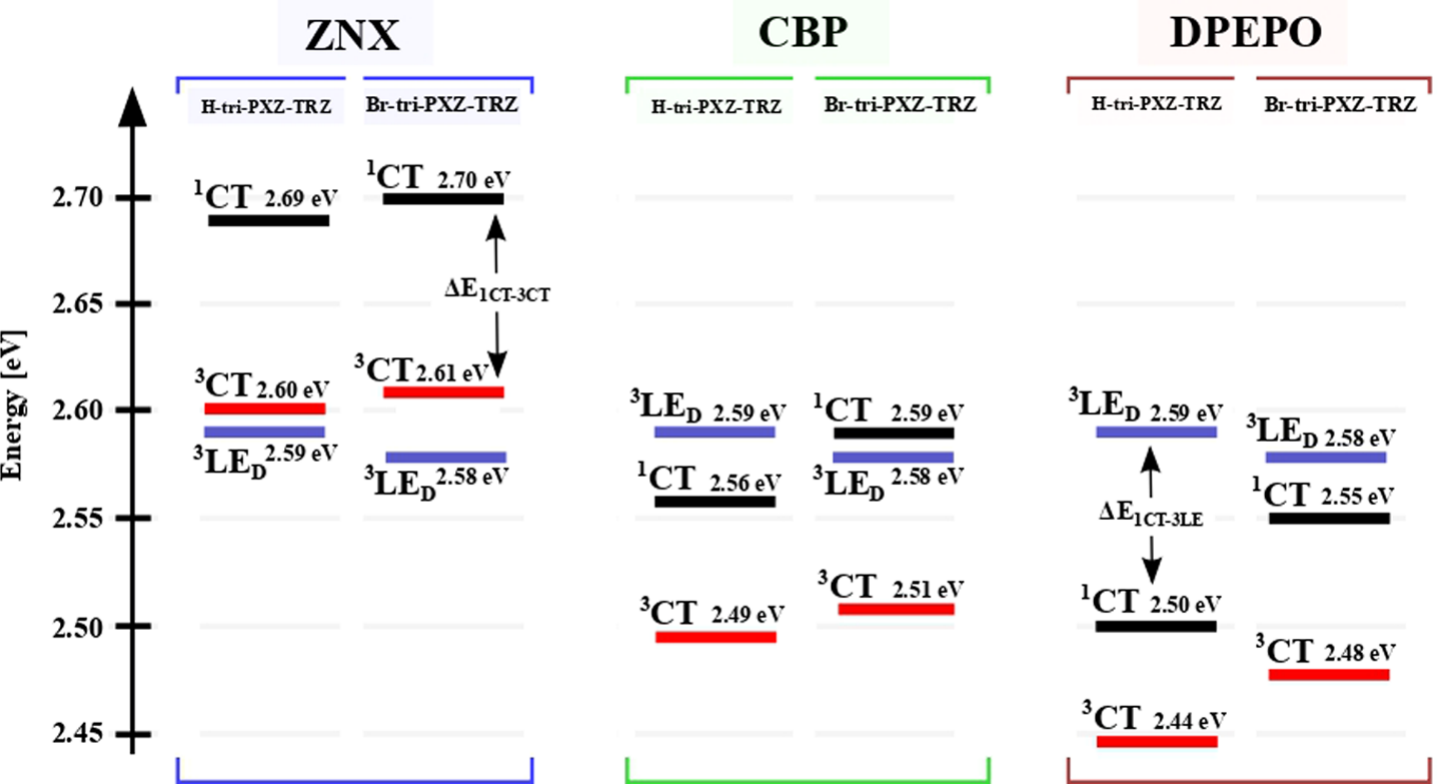
Energy-level diagram of the excited states of investigated emitters in various media.

Based on the steady-state PL spectra and respective phosphorescence measurements for **H-tri-PXZ-TRZ** (Figure S4A–C) and **Br-tri-PXZ-TRZ** (Figure S4D–F), the alignment of the lowest excited states in ZNX, CBP, and DPEPO was determined (Figure 2). Because the excited states of LE character are much less sensitive to polarity than the CT ones, the energies of the ^3^LE_D_ states in **H-tri-PXZ-TRZ** and **Br-tri-PXZ-TRZ** are assumed to remain at the same level in all of the studied media. As the polarity increases, the energy gap Δ*E*_^1^CT–^3^LE_ between the ^1^CT and ^3^LE_D_ states in **H-tri-PXZ-TRZ** decreases: Δ*E*_^1^CT–^3^LE_ = 0.10, −0.04, and −0.09 eV in ZNX, CBP, and DPEPO, respectively. In the case of **Br-tri-PXZ-TRZ**, this trend is also observed, but the respective values of Δ*E*_^1^CT–^3^LE_ are slightly higher due to the blue-shifted ^1^CT states and red-shifted ^3^LE_D_: Δ*E*_^1^CT–^3^LE_ = 0.12, 0.01, and −0.03 eV in ZNX, CBP and DPEPO, respectively. The energy gaps Δ*E*_^1^CT–^3^LE_ between the ^1^CT and ^3^CT states were determined to be minimal in the medium of highest polarity, namely, the DPEPO films: Δ*E*_^1^CT–^3^LE_ = 0.06 and 0.07 eV for **H-tri-PXZ-TRZ** and **Br-tri-PXZ-TRZ**, respectively. Higher values were observed in the CBP films: Δ*E*_^1^CT–^3^LE_ = 0.07 and 0.08 eV, respectively. The energies of the ^3^CT states in ZNX were found by extrapolation of the linear dependence of Δ*E*_^1^CT–^3^LE_ on the ^1^CT energy, observed in CBP and DPEPO, as can be seen in Figure S5. Thus, the obtained values of Δ*E*_^1^CT–^3^LE_ are 0.09 and 0.10 eV for **H-tri-PXZ-TRZ** and **Br-tri-PXZ-TRZ**, respectively.

According to previous studies of triphenyl-*s*-triazine emitters, the ^3^LE_A_ state localized on such an acceptor should have energy above 2.83 eV. It is more than 0.2 eV above the lowest triplet states in all investigated media; thus, in such a case, the ^3^LE_A_ state cannot significantly affect the dynamics of rISC.

### Time-Resolved PL Measurements

The time-resolved emission spectroscopy (TRES) spectra in Figure S6 evidence that, in all studied media, both emitters exhibit TADF. The observed spectral red and blue shifts at different time delays originate from the coexistence of rotational isomers with different torsion angles between the donor and acceptor planes, as was revealed previously by numerous experimental and theoretical analyses.^8,24^

**Figure 3:**
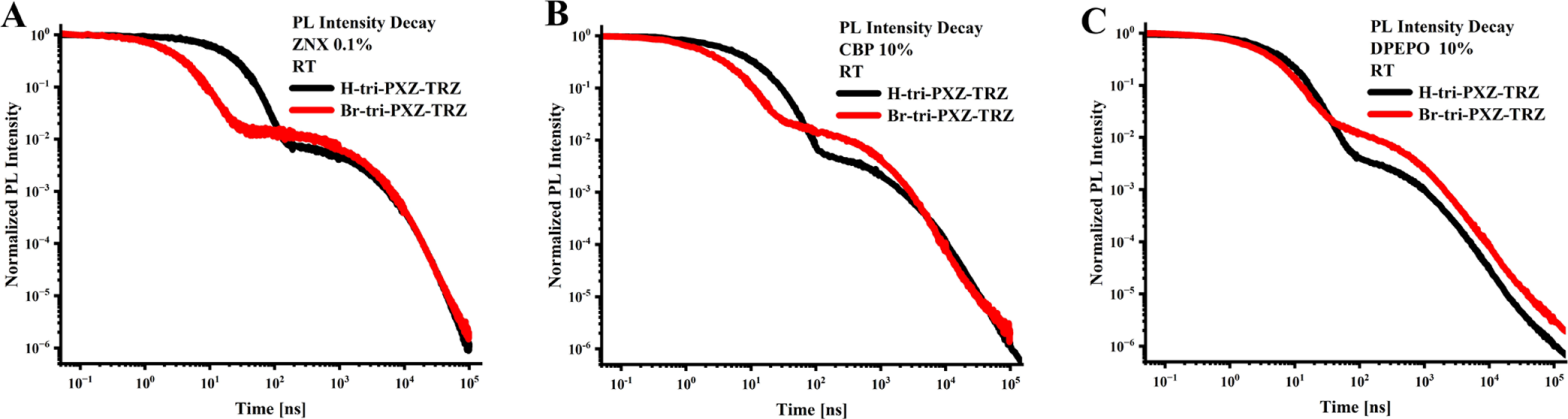
PL decays of **H-tri-PXZ-TRZ** and **Br-tri-PXZ-TRZ** in 0.1% ZNX (A), 10% CBP (B), and 10% DPEPO (C). Conditions: room temperature (RT), vacuum, and excitation wavelength = 330 nm.

Figures 3, S7, and S8 present the PL intensity decays of **H-tri-PXZ-TRZ** and **Br-tri-PXZ-TRZ** with well-distinguished time domains of prompt (PF) and delayed (DF) fluorescence. In all media, the brominated derivative shows a significant reduction of the PF lifetime (*τ*_PF_) caused mainly by the acceleration of ISC (*k*_ISC_; Table 2). The rate of ^1^CT radiative deactivation (*k*_r_) of **Br-tri-PXZ-TRZ** (*k*_r_ = 2.6, 2.4, and 2.1 × 10^7^ s^–1^ in ZNX, CBP, and DPEPO, respectively) is slightly higher than that of **H-tri-PXZ-TRZ** (*k*_r_ = 2.4, 2.2, and 1.9 × 10^7^ s^–1^ in the respective hosts), which can be related to the electron-withdrawing effect of the halogen in **PXZ**. The introduction of bromine does not affect the nonradiative deactivation channel (*k*_nr_) from the S_1_ state, and the *k*_nr_ value remains the same for both emitters.

**Table 2:** Lifetimes and Rate Constants of Photophysical Processes

cmpd	medium	Φ_DF_/Φ_PF_	*τ*_PF_^a^ [ns]	*τ*_DF_^a^ [μs]	*k*_r_ [10^7^ s^–1^]	*k*_nr_ [10^7^ s^–1^]	*k*_ISC_ [10^7^ s^–1^]	*k*_rISC_ [10^6^ s^–1^]
**H-tri-PXZ-TRZ**	ZNX	1.1	19.2 ± 0.9	5.0 ± 0.3	2.3	0.1	2.9	0.44
**Br-tri-PXZ-TRZ**		8.9	4.7 ± 0.3	3.9 ± 0.4	2.6	0.2	20.8	2.26
**H-tri-PXZ-TRZ**	CBP	0.8	15.3 ± 0.7	8.2 ± 0.5	2.2	1.4	2.6	0.22
**Br-tri-PXZ-TRZ**		3.4	5.5 ± 0.5	2.1 ± 0.3	2.4	1.6	13.7	1.75
**H-tri-PXZ-TRZ**	DPEPO	0.2	14.2 ± 0.8	15.8 ± 0.8	1.9	4.3	0.7	0.07
**Br-tri-PXZ-TRZ**		1.3	6.0 ± 0.6	6.3 ± 0.3	2.1	4.5	9.3	0.49

a The details of error calculations are included in Section S1.

In all media in the DF regime of **Br-tri-PXZ-TRZ**, a notable reduction of the *τ*_DF_ can be observed thanks to the accelerated reverse intersystem crossing (*k*_rISC_). In CBP, the shortest value of *τ*_DF_ = 2.1 μs was observed for the brominated emitter. The highest value of *k*_rISC_ of 2.3 × 10^6^ s^–1^ was determined for **Br-tri-PXZ-TRZ** in ZNX, which is 8.3 times faster than that of **H-tri-PXZ-TRZ** (*k*_rISC_ = 0.44 × 10^6^ s^–1^). It is worth noticing that, in ZNX films containing **Br-tri-PXZ-TRZ**, the ratio of DF and PF quantum yields is impressively large (Φ_DF_/Φ_PF_ ∼ 9), while 90% of the emission originates from DF thanks to the crucial role of ISC (*k*_ISC_/*k*_r_ ∼ 8) in deactivation of the ^1^CT state and the relatively low ratio *k*_ISC_/*k*_rISC_ ∼ 90. Concerning the effect of a medium on the two main photophysical values, *k*_r_ and *k*_rISC_, one can notice that both emitters show a decrease of these rate constants as the polarity increases. The dependence of *k*_r_ on the polarity is usually observed in the D–A systems^25^ and is related to the oscillator strength *f*_^1^CT–S_0__. In polar media, stabilization of the CT states lead to a more effective separation of frontier orbitals involved in the S_1_–S_0_ transition, which reduces *f*_^1^CT–S_0__ and, therefore, *k*_r_, as described by the Sticker–Berg law.^26,27^

### “Hyperfluorescence”

To date, most of the best TADF emitters are based on the D–A architecture. Although the D–A design strategy provides minimal Δ*E*_ST_ and high rISC rates, it sacrifices the probability of radiative deactivation to ground state S_0_. Apparently, in an ideal emitter, the radiative rate constant should be as high as possible. Another important consequence of the presence of a D–A scaffold is a broad emission spectrum, typical for the ^1^CT–S_0_ transition. However, to meet the requirements of OLED display applications, the emission spectrum should be narrow to ensure high color purity.

**Figure 4:**
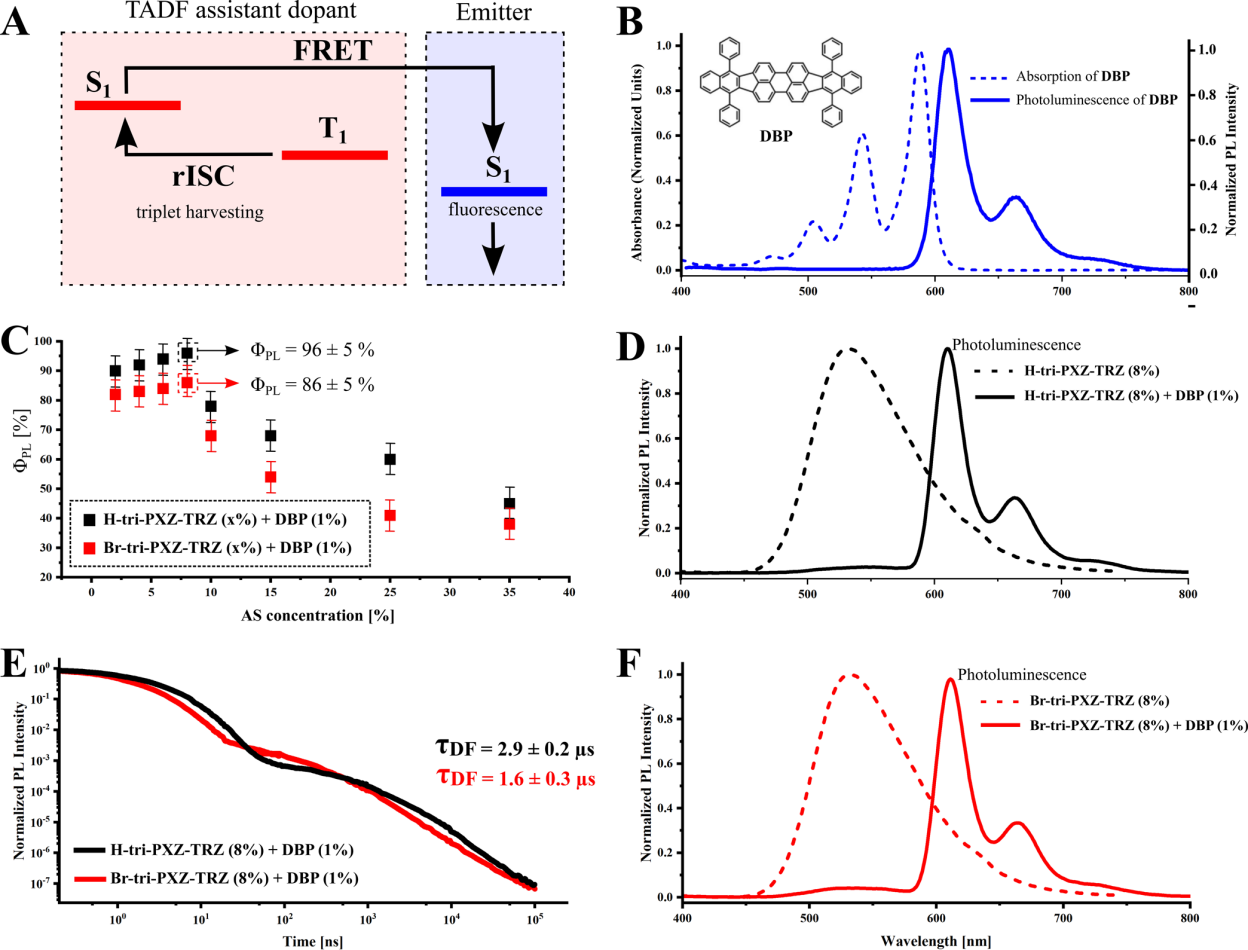
Photophysical characterization of systems. (A) Schematic illustration of the HF mechanism. (B) Absorption (in CHCl_3_) and steady-state emission (1% in CBP) of DBP. (C) PLQY values as a function of the TADF assistant dopant (AS) concentration within the 3–35% range with a fixed concentration of 1% DBP in the CBP host. (D) PL spectra of **H-tri-PXZ-TRZ** and the mixed film 8% **H-tri-PXZ-TRZ** with 1% DBP in CBP. (E) PL intensity decays of - based films integrated over the DBP emission band (610–650 nm) measured at room temperature using excitation wavelength *λ*_exc_ = 330 nm. (F) PL spectra of **Br-tri-PXZ-TRZ** and the mixed film 8% **Br-tri-PXZ-TRZ** and 1% DBP in CBP.

The HF approach has been proposed as a solution of the above-mentioned problems.^18^ In principle, a typical system consists of two dopants: (i) a TADF emitter and (ii) a conventional fluorescent molecule acting as a terminal emitter. The TADF emitter is responsible for triplet harvesting thanks to its small Δ*E*_ST_ and efficient rISC. On the other hand, the fluorescent molecule with a preferably rigid and planar structure serves as an effective emissive entity with high *k*_r_ (∼10^9^ s^–1^) and an intrinsically narrow band emission. In such a system, the excitation energy is transferred from the TADF molecule to a terminal emitter via Förster resonance energy transfer (FRET; Figure 4A), which is achieved by adjusting the spectral overlap between fluorescence of a TADF emitter and absorption of a conventional fluorescent molecule. The HF approach not only improves the color purity but also enhances the stability of all-organic OLEDs.^28^

Because of significant acceleration of rISC in **Br-tri-PXZ-TRZ** and the appropriate maximum of PL spectra *λ*_max_ = 530 nm (8% CBP), we tested this TADF emitter as an assistant dopant with the popular red fluorophore DBP as the terminal emitter with the maximum of emission *λ*_max_ = 610 nm (1% DBP in CBP, w/w). As is shown in Figure 4B,D,F, the PL spectra of **H-tri-PXZ-TRZ** and **Br-tri-PXZ-TRZ** perfectly overlap with the absorption band of DBP, therefore meeting the energetic criterion of FRET. In fact, the PL spectrum of a film containing 8% of **Br-tri-PXZ-TRZ** and 1% DBP in the CBP host matrix evidences effective quenching of the emission of TADF emitters around 540 and 530 nm and domination of the DBP emission band. Figure 4C shows the results of optimization of the components’ concentrations of both systems. The highest PLQY values were obtained in the case of 8% TADF molecule and 1% DBP in the CBP host: Φ_PL_ = 95 and 86% for **H-tri-PXZ-TRZ** and **Br-tri-PXZ-TRZ**, respectively (Table 3). Higher concentrations of TADF assistant dopants lead to a decrease of the PLQY values. Figure 4E presents PL intensity decays of optimized HF systems. Most importantly, the use of **Br-tri-PXZ-TRZ** affords a reduction of *τ*_DF_ from 2.9 to 1.6 μs, while Φ_PL_ remains above 85%. Most DF occurs in the submicrosecond domain (Figure 4E), which indicates a high applicative potential of **Br-tri-PXZ-TRZ** as a component of emissive layers of OLEDs.

**Table 3:** Comparison of the Photophysical Properties of the HF Systems

	[TADF em.], %^a^	[DBP], %^a^	Φ_PL_,^b^ %	*τ*_PF_,^c^ ns	*τ*_DF_,^b^^c^ μs
**H-tri-PXZ-TRZ**	8	1	95 ± 5	2.9 ± 0.1	2.9 ± 0.2
**Br-tri-PXZ-TRZ**	8	1	86 ± 5	2.7 ± 0.1	1.6 ± 0.3

a Concentration of TADF compound and DBP emitter.

b PLQYs determined with a ±5% error, according to the equipment specifications.

c The details of error calculations are included in Section S1.

### EL Performance

To check whether the enhanced rISC and shortened DF lifetime of the emissive layer can, in fact, improve the performance of OLEDs, several devices were fabricated. We focused on further optimization of the emissive layer, which plays a crucial role in determining the device’s efficiency and color purity. The impact of varying the concentrations of two TADF emitters, **H-tri-PXZ-TRZ** and **Br-tri-PXZ-TRZ**, and a terminal emitter, DBP, was examined. A total of 14 structures were fabricated using solution process techniques and different concentrations of the TADF emitter and DBP (Figures S9 and S10). The devices were characterized using current density–voltage (*J*–*V*) measurements and EL spectroscopy. The *J*–*V* measurements revealed that the devices exhibited a nonlinear current–voltage relationship, indicating that the charge-injection and -transport processes were efficient. In spite of the fact that the external quantum efficiency (EQE) of such devices ranged from 1% to 5% depending on the material composition, much lower than previously presented evaporated devices,^18^ such tests allowed us to find an optimal emissive layer regarding EL parameters (Figures S9 and S10). The EL spectra of the devices exhibited characteristic emission peaks corresponding to the TADF emitter and assistant dopant. The shape of the EL spectra was affected by the composition of the emissive layer, with 3% DBP and 30% TADF emitter (w/w) devices exhibiting the purest emission spectra of the terminal emitter, devoid of additional bands of the TADF emitter.

**Figure 5:**
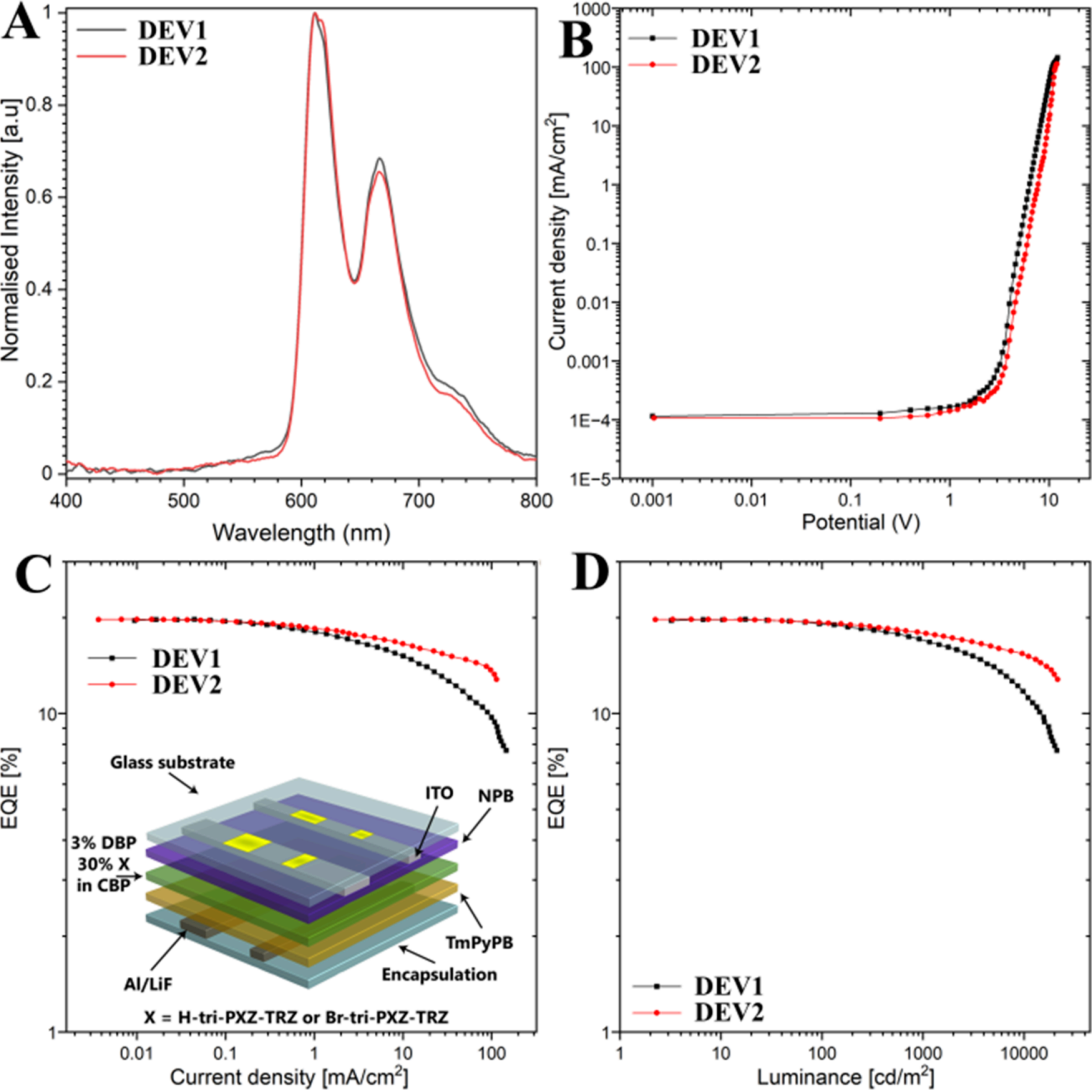
Characteristics of the vacuum-deposited HF OLED devices. (A) EL spectra. (B) *IV* characteristic. Dependence of EQE on the current density (C) and luminance (D).

Based on these results, the fully evaporated devices were investigated (Figure 5). For both compounds, the following optimal device structure was obtained: ITO/NPB (40 nm)/3% DBP, 30% **H-tri-PXZ-TRZ** (DEV1) or **Br-tri-PXZ-TRZ** (DEV2) in CBP (30 nm)/TmPyPB (40 nm)/LiF (1 nm)/Al (100 nm) (Figure 5). Figure 5A shows the EL spectra of both devices, providing insights into their emission wavelength and color characteristics. The devices exhibit dual peak emission wavelengths of around 610 and 665 nm from the DBP terminal emitter, corresponding to red emission. Figure 5B presents the current density–bias (*IV*) characteristics of both devices, revealing their nonlinear operation. The current density increases with increasing applied voltage, but the rate of increase decreases at higher voltage levels. This characteristic behavior is attributed to the saturation of charge-carrier transport within the device. The *IV* curves of both devices exhibit a similar trend, indicating comparable charge-carrier-transport dynamics. Figure 5C compares the EQE curves of **H-tri-PXZ-TRZ** and **Br-tri-PXZ-TRZ**, providing a measure of their efficiency of conversion of electrical energy into light. EQE represents the percentage of injected electrons that recombine radiatively emitting photons. The **H-tri-PXZ-TRZ** OLED achieves a EQE_max_ of 20.1%, while the **Br-tri-PXZ-TRZ** OLED reaches a slightly lower EQE_max_ of 19.5%.

Figure 5D presents the EQE–luminance (EQE–L) characteristics of **H-tri-PXZ-TRZ**- or **Br-tri-PXZ-TRZ**-based devices, illustrating the relationship between EQE and luminance. Luminance is a measure of the brightness of the emitted light. Both devices exhibit an initial increase in EQE with increasing luminance, followed by a decrease at higher luminance levels (efficiency roll-off). The highest luminance was observed for the **Br-tri-PXZ-TRZ** device with value up to 21511 cd/m^2^ (Table 4). Moreover, the maximum luminance efficiency (η_max_) was observed for the **Br-tri-PXZ-TRZ** device, which exhibited better light extraction at lower current with values up to 24.81 cd/A. This behavior arises from the competition between the radiative and nonradiative recombination processes. As the luminance increases, the probability of nonradiative recombination processes also increases, leading to the EQE roll-off. A comparative analysis of the electrooptical characteristics of the **H-tri-PXZ-TRZ** and **Br-tri-PXZ-TRZ** devices reveals some notable distinctions. While **H-tri-PXZ-TRZ** holds a slight advantage in terms of EQE_max_, **Br-tri-PXZ-TRZ** exhibits superior stability. Namely, at 100 cd/m^2^, the EQE roll-off is negligibly small at −1%, while at 1000 and 10000 cd/m^2^, this value does not exceed 8% and 21%, respectively (Table 4). In contrast, the device comprising **H-tri-PXZ-TRZ** exhibits from 2–4 times higher roll-off. This suggests that **Br-tri-PXZ-TRZ** may be more suitable for applications requiring long-term operation and consistent light output.

**Table 4:** EL Data of the Vacuum-Deposited HF OLED Devices^a^

						EQE (%)/*roll-off* (%)^*d*^			
device	*V*_ON_ [V]	EL_max_ [nm]	*L*_max_ [cd/m^2^]	η_max_ [cd/A]	CIE coord. (*x*, *y*)	max	100 cd/m^2^	1000 cd/m^2^	10000 cd/m^2^
DEV1 (**H-tri-PXZ-TRZ**)	2.5	610, 665	21071	19.38	0.645, 0.340	20.08	19.12/*4.8*	17.02/*15.2*	11.64/*42.0*
DEV2 (**Br-tri-PXZ-TRZ**)	3.0	610, 665	21511	24.81	0.645, 0.336	19.51	19.29/*1.1*	17.91/*8.2*	15.34/*21.4*

a The roll-off was calculated as 100(EQE – EQE_max_)/EQE_max_.

The improved stability and lower roll-off of the **Br-tri-PXZ-TRZ** device can be attributed to several factors. Most likely, the key factor is the higher rISC rate constant in **Br-tri-PXZ-TRZ** with a pronounced HA effect, which helps to successfully compete with nonradiative deactivation processes contributing to the enhanced stability of the device. Furthermore, due to decreased HOMO energy, the **Br-tri-PXZ-TRZ**-based device can incorporate better HILs and HTLs, which can enhance the device’s ability to efficiently transport charges and prevent recombination losses. Additionally, the choice of materials and their interfaces may play a significant role in reducing nonradiative recombination processes and improving the device’s overall stability.

A slightly lower EQE_max_ of **DEV 2** most likely results from a 9% lower PLQY of the CBP–**Br-tri-PXZ-TRZ**–DBP system (Table 3). Interestingly, the differences in the PLQYs of both emitters in the host–TADF emitter systems without DBP does not exceed 2–4 absolute percent within the range of experimental error (Table 1) and thus can be regarded as negligible. This is in spite of the fact that, under the introduction of bromine, the calculated SOC for the T_1_–S_0_ transition increases by almost twice as much from 1.59 to 3.82 cm^–1^. This indicates that, in the triplet state of **Br-tri-PXZ-TRZ**, nonradiative relaxation to the ground state is still too slow and cannot compete with rISC and further deactivation of S_1_ via fluorescence or FRET. Therefore, a minor negative influence of HAs appears in the presence of a terminal emitter, DBP, and/or during the FRET process. Within the collected data and available correlations, we assume that **Br-tri-PXZ-TRZ** can accelerate ISC and triplet deactivation in the DBP molecule by an external HA effect, which is supported by previously analyzed mechanisms of energy losses in hyperfluorescent systems.^29^ However, as mentioned above, regarding EQE_max_, this has a minor effect on the device performance, while with regard to EQE roll-off, its role is completely negligible.

The decrease of the EQE roll-off while maintaining the high EQE_max_ of the OLED device is one of the key requirements for potential display or lightning applications of organic emitters. Previously reported OLEDs based on brominated emitters showed worse stability compared to the reference emitters.^30−32^ To the best of our knowledge, the improvement discussed here of the device stability with practically stable EQE_max_ is the first example of the successful application of nonmetallic HAs of row 4 of the periodic table like bromine in the emissive layer of the OLED.

### Quantum-Chemical Calculations and the TADF Mechanism

To understand the origin of TADF, the superior EL behavior of **Br-tri-PXZ-TRZ**, and the mechanism of rISC acceleration by the HA effect, the electronic properties of the emitters were investigated with the help of (TD-)DFT calculations. The molecular orbital analysis conducted in optimized geometries confirmed that the lowest excited singlet and triplet states are of CT nature (Figure S11). Due to the presence of three donor units in both emitters of *C*_3_ symmetry), the ^1^CT and ^3^CT states, as well as ^3^LE_D_, are three times degenerated. However, to simplify calculations, only one of each three existing ^1^CT, ^3^CT, and ^3^LE_D_ states was considered, which is justified further.

In order to verify whether the ^3^CT → ^1^CT or ^3^LE_D_ → ^1^CT pathway is responsible for the rISC acceleration observed in **Br-tri-PXZ-TRZ**, the rate constants *k*_^3^CT→^1^CT_ and *k*_^3^LE→^1^CT_ were calculated by means of the DFT level of theory using the Marcus–Hush equation^6,38^ (for detailed procedures of calculations, see the SI).

#### rISC: ^3^CT → ^1^CT Transition

**Figure 6:**
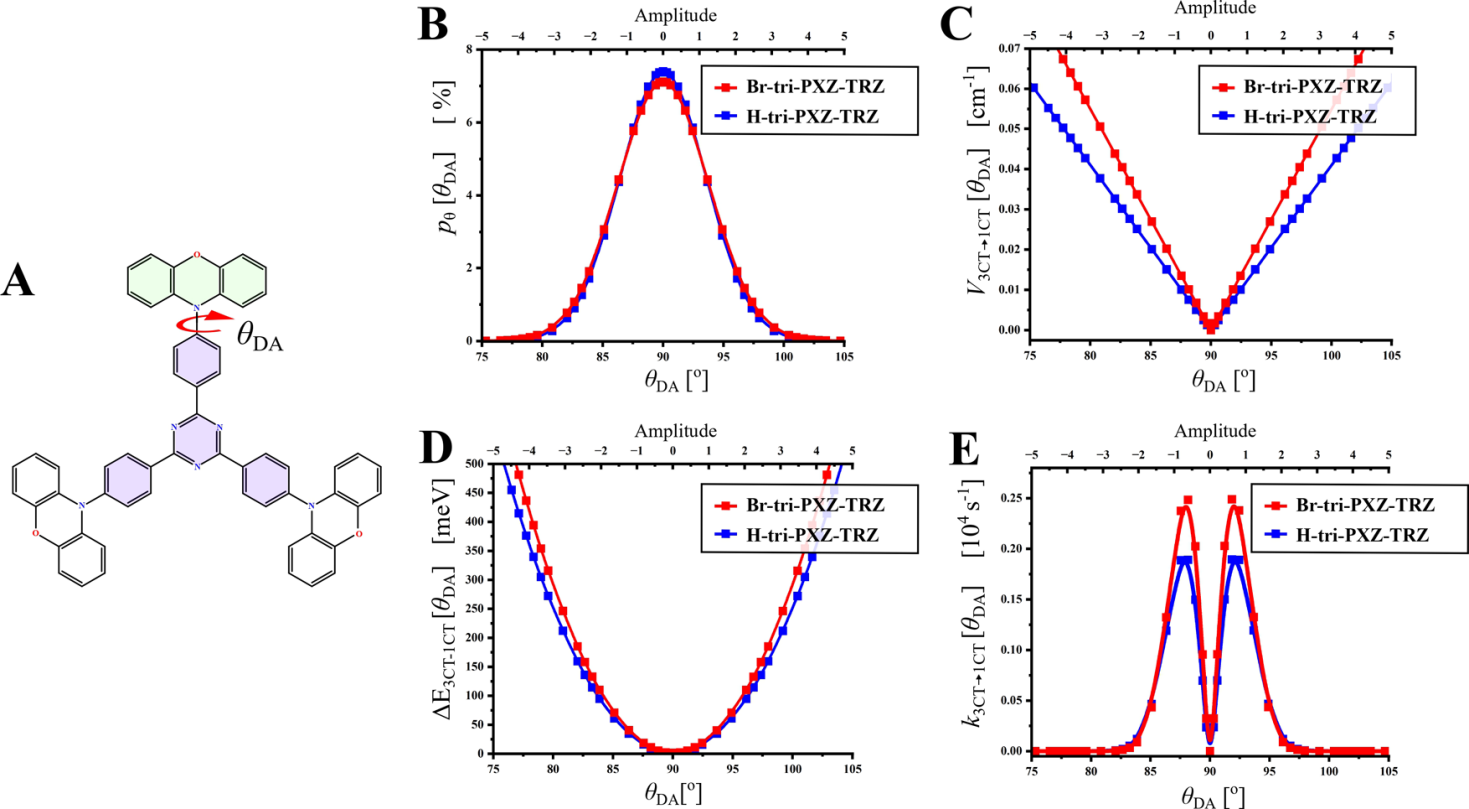
(A) Structure of **H-tri-PXZ-TRZ** with highlighted dihedral angle *θ*_DA_. (B) Calculated population *p*_θ_[*θ*_DA_] from the Boltzmann distribution. (C) SOC constant values *V*_^3^CT→^1^CT_[*θ*_DA_]. (D) Energy gaps Δ*E*_^1^CT–^3^CT_[*θ*_DA_]. (E) Individual constant rates *k*_^3^CT→^1^CT_[*θ*_DA_] of *θ*_DA_ rotamers.

According to the El Sayed selection rules, the ^3^CT → ^1^CT transition is forbidden due to the lack of change of the orbital momentum.^4^ In the optimized geometry of studied compounds, the dihedral angle(s) *θ*_DA_ between the donor(s) and acceptor is (are) close to 90°, and the calculated SOC constant between pure CT states (*V*_^3^CT→^1^CT_ in eq S11), in fact, equals zero (Figure 6A,C). However, our previously developed theoretical models^33,34^ of the TADF mechanism in D–A emitters evidenced that SOC of the ^3^CT → ^1^CT transition can be effectively activated by the low-frequency molecular motions. Specifically, it was demonstrated that there is a strong dependence of *V*_^3^CT→^1^CT_ on the dihedral angle (*θ*_DA_) between donor and acceptor fragments. Consequently, the vibrations causing the change of *θ*_DA_ can activate the ^3^CT → ^1^CT channel of rISC. It should be noted that the *θ*_DA_ vibration is a dynamic process in low-viscosity media such as liquid solutions, while in amorphous matrixes, the increased energy barrier for *θ*_DA_ rotation leads to the coexistence of specified rotational isomers (*θ*_DA_ rotamers), whose population is described by the statistical Boltzmann distribution function *p*_θ_[*θ*_DA_].

To analyze the contribution of the ^3^CT → ^1^CT channel in rISC of the studied emitters, the following calculations were performed. Figure 6 presents the dependencies of the *p*_θ_[*θ*_DA_] population (Figure 6B), SOC constant values *V*_^3^CT→^1^CT_ (Figure 6C), energy gaps Δ*E*_^1^CT–^3^CT_ (Figure 6D), and individual rate constants *k*_^3^CT→^1^CT_ (Figure 6E) on the dihedral angle *θ*_DA_. For clarity, the horizontal axis is also expressed as the amplitude of the *θ*_DA_ vibration. The calculated statistical sums of *k*_^3^CT→^1^CT_ of various *θ*_DA_ rotamers (∑*p*_θ_*k*_3CT→1CT_) equal 0.95 × 10^3^ and 1.2 × 10^3^ s^–1^ for **H-tri-PXZ-TRZ** and **Br-tri-PXZ-TRZ**, respectively (for the detailed calculation procedure, see the SI). Such low values originate from relatively small changes in *V*_^3^CT→^1^CT_ upon the *θ*_DA_ vibration within the 90 ± 5° range of the highest probability of *θ*_DA_ rotamers: in both emitters, the calculated statistical value of *V*_^3^CT→^1^CT_ (∑ *p*_θ_*V*_^3^CT→^1^CT_) does not exceed 0.01 cm^–1^. The theoretically obtained rISC rate constants are 2 orders of magnitude lower than the experimental ones (Table 2), which together with the small difference in *k*_^3^CT→^1^CT_ between studied compounds indicate that the ^3^CT → ^1^CT transition plays a negligible role in rISC of the discussed emitters.

#### rISC: ^3^LE_D_ → ^1^CT Transition

**Figure 7:**
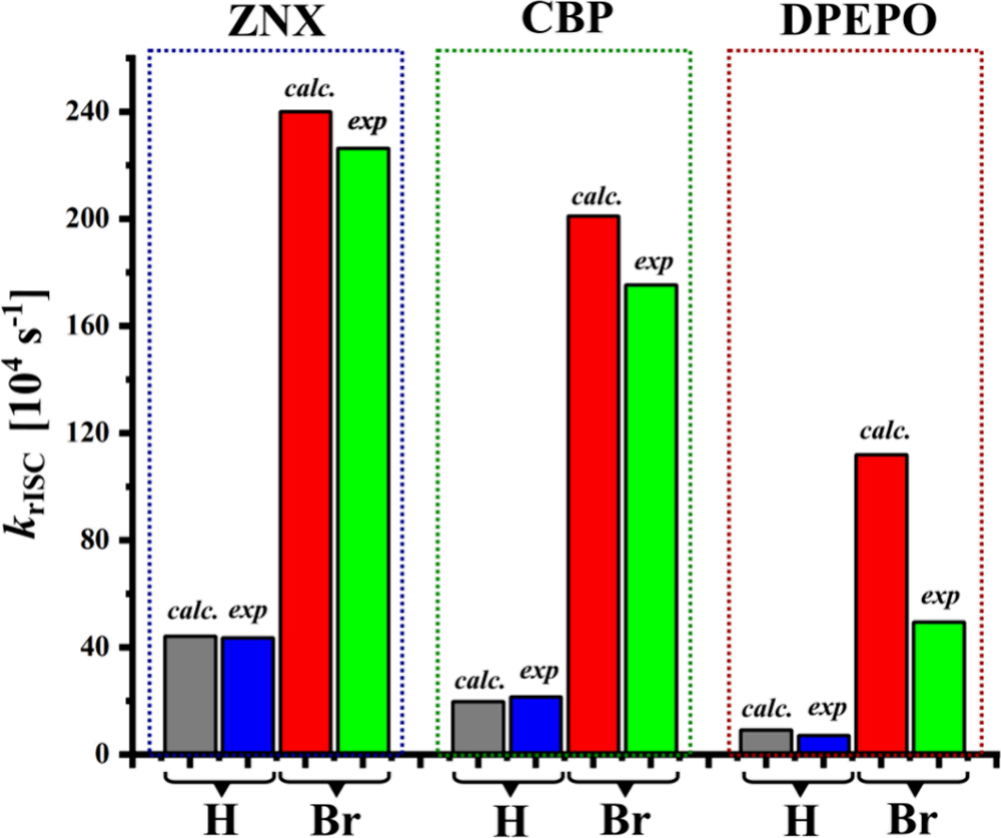
Comparison of the experimental *k*_rISC_ and theoretically predicted rate constants of the ^3^LE_D_ → ^1^CT transition multiplied by the population of the ^3^LE_D_ state (χ_^3^LE_*k*_^3^LE→^1^CT_).

In contrast to this, analysis of the ^3^LE_D_ → ^1^CT transition revealed that this pathway clearly dominates in the rISC process. The energetic closeness of ^1^CT to the ^3^LE_D_ state localized on the nonbrominated/brominated phenoxazine donor and relatively high SOC constant values *V*_^3^LE→^1^CT_ (0.88 and 2.67 cm^–1^ for **H-tri-PXZ-TRZ** and **Br-tri-PXZ-TRZ**, respectively) enables efficient rISC. This is in spite of the low population of excited molecules in ^3^LE_D_; for instance, in CBP, such χ_^3^LE_ populations are 1.7% and 5.5%, respectively (Table S5). Table 5 demonstrates the calculated parameters and constant rates of the ^3^LE_D_ → ^1^CT transition. The experimentally observed decrease of *k*_rISC_ when moving from ZNX to DPEPO (Figure 7) can be explained by the decrease of the ^3^LE_D_-state population χ_^3^LE_ in favor of the ^3^CT one (Figure 2) due to their different sensitivities to the polarity of these states. A more polar environment stabilizes the ^3^CT state, leading to domination of the slow ^3^CT → ^1^CT transition, which in the studied emitters cannot compete effectively with nonradiative deactivation.

**Table 5:** Calculated Parameters and Constant Rates of the ^3^LE_D_ → ^1^CT Transition

cmpd	medium	Δ*E*_^1^CT–^3^LE_ [eV]	λ_^3^LE→^1^CT_ [eV]	*V*_^3^LE→^1^CT_ [cm^–1^]	*k*_^3^LE→^1^CT_ [10^6^ s^–1^]	χ_^3^LE_ [%]	χ_^3^LE_*k*_^3^LE→^1^CT_ [10^6^ s^–1^]
**H-tri-PXZ-TRZ**	ZNX	0.10	0.24	0.88	0.79	55.7	0.44
**Br-tri-PXZ-TRZ**		0.12	0.28	2.67	3.60	66.7	2.40
**H-tri-PXZ-TRZ**	CBP	–0.04	0.24	0.88	11.6	1.7	0.19
**Br-tri-PXZ-TRZ**		0.01	0.28	2.67	36.8	5.5	2.02
**H-tri-PXZ-TRZ**	DPEPO	–0.09	0.24	0.88	32.0	0.3	0.09
**Br-tri-PXZ-TRZ**		–0.03	0.28	2.67	82.0	1.5	1.13

### Connection with Other Investigations

From the point of view of the TADF mechanism, the investigations described here represent an important example of (1) a weak ^3^CT–^1^CT interaction in a multiple D–A emitter and (2) efficient acceleration of the ^3^LE → ^1^CT transition without an increase of the nonradiative deactivation rate. The first issue is rather unexpected for two reasons. On the one hand, TADF of several blue emitters comprising the 9,9-dimethyl-9,10-dihydroacridine (DMAC) donor as well as its brominated derivatives with various acceptors including the *s*-triazine one follow the two-state theoretical models for D–A-type TADF emitters.^8,9,35^ It was concluded that rISC in such TADF emitters is driven by the direct SOC between the ^1,3^CT states. Moreover, in such emitters, the HA effect induced by bromine(s) enhanced SOC and thus the rate of the ^3^CT → ^1^CT transition thanks to the specific vibrations involving bromine atoms. The direct two-state TADF mechanism in such blue emitters revealed that the contribution of the ^3^LE_D_ → ^1^CT transition in the rISC process is secondary or negligible in most of media, while the key parameter limiting the rISC rate is the energy gap Δ*E*_^1^CT–^3^CT_. Taking into account the almost 10 times smaller Δ*E*_^1^CT–^3^CT_ values predicted for the optimal geometries of **tri-PXZ-TRZ** derivatives, reaching 1 meV (Tables S3 and S4) compared to 7–12 meV for analogues with a weaker DMAC donor,^8,29^ one should thus expect a more efficient ^3^CT → ^1^CT transition, which is not the case. In light of the results presented in this paper, an important question arises, *Why, in the case of the TADF emitters based on stronger and multiple D–A scaffold, is the presence of the^3^LE*_*D*_*state but not the reducedΔE_^1^CT–^3^CT_value responsible for the observed fast rISC?*

Within the three-state TADF models, which take into account second-order SOC effects and the spin-vibronic mechanism of the ^1,3^CT and ^3^LE interaction,^36−38^ the presence of several donor and/or acceptor units is supposed to favor faster rISC due to the ^3^LE level degeneracies and increased density of states and possible pathways within which rISC can be realized.^7,39^ According to the El Sayed rules, SOC is larger and (r)ISC is faster when the change of spin is accompanied by a large change in the orbital angular momentum.^40^ One can thus suggest that, when rISC is realized via the ^3^LE → ^1^CT channel, multiplication of the ^3^LE states in one electronic system should provide further improvement of the TADF parameters.

**Scheme 2:**
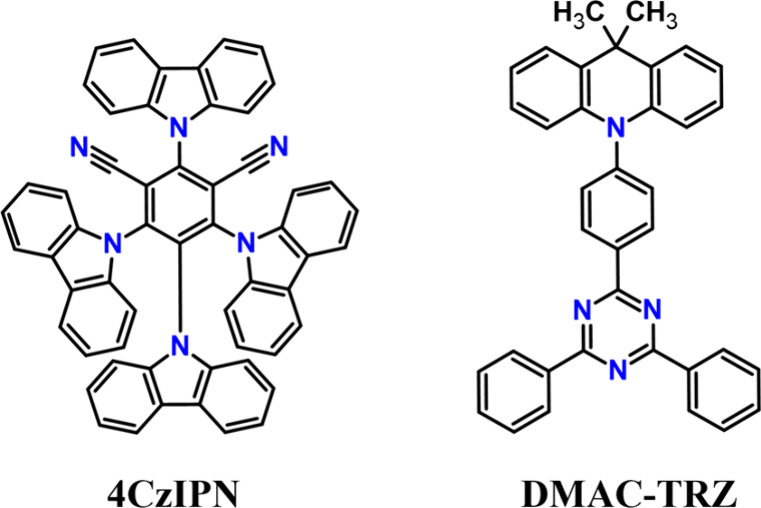
Structures of Previously Investigated Emitters

One of the most representative examples of such multiple D–A TADF emitters is **4CzIPN**(41) with four carbazole donors (Scheme 2). In this case, several different combinations of ^1,3^CT states are formed, depending on which donor is involved in the formation of the respective ^1,3^CT excited states. Although all four donor units are identical, several ^3^CT states have different nature and energy due to structural differences, namely, different *θ*_DA_ dihedral angles in partial molecular structures. Importantly, even partial planarization of some of *θ*_DA_ increases the conjugation and contribution of the LE nature in some of the lowest excited triplet states, resulting in various ^3^LE/^3^CT mixed states with increased SOC of T_*n*_–S_1_.^42^ It should be also noted that there is no degeneracy of the ^1,3^CT states in such systems.

Much less pronounced ^3^LE/^3^CT mixing was also observed in the D–A emitters with a DMAC donor of similar strength but bulkier size than those of carbazole. In fact, in **DMAC-TRZ** (Scheme 2),^8,33,34^ a minimal increase of the overlap of HOMO and lowest unoccupied molecular orbital (LUMO) during the *θ*_DA_ rotation explains the activation of the ^3^CT → ^1^CT SOC. Compared to **4CzIPN** and other carbazole derivatives, a larger size of the DMAC donor causes the restriction of such rotations, resulting in a *θ*_DA_ distribution within 90 ± 25°.

The cases of **H-tri-PXZ-TRZ** and **Br-tri-PXZ-TRZ** are different mainly due to the much stronger phenoxazine donor. Together with its large size, this causes efficient stabilization of CT, and according to the thermodynamics of rotamers (Tables S3 and S4), strongly reduces the *θ*_DA_ distribution to 90 ± 10° and thus further decreases the ^3^LE/^3^CT mixing and ^3^CT–^1^CT SOC enhancement. In spite of a sufficient decrease of the energy gap Δ*E*_^1^CT–^3^CT_ and reorganization energy *λ*_^3^CT→^1^CT_, the SOC constant in the accessible *θ*_DA_ rotamers is too low to enable *k*_^3^CT→^1^CT_ above 3 × 10^3^ s^–1^.

It should be noted that partial molecular structures composed of single donor **PXZ** and **TRZ** units in the optimized geometries of **H-tri-PXZ-TRZ** and **Br-tri-PXZ-TRZ** have identical structural parameters, including the dihedral angles *θ*_DA_ equal 90° (Figure S11). This is the reason for the high degeneracy of the ^3^CT states without a detectable LE contribution. The SOC between such degenerated (3 × ^3^CT)–(3 × ^1^CT) states does not exceed 0.08 cm^–1^ (Table S7). In the studied cases, the spin-flip transitions thus can be referred to as pure ^3^CT → ^1^CT.

**Figure 8:**
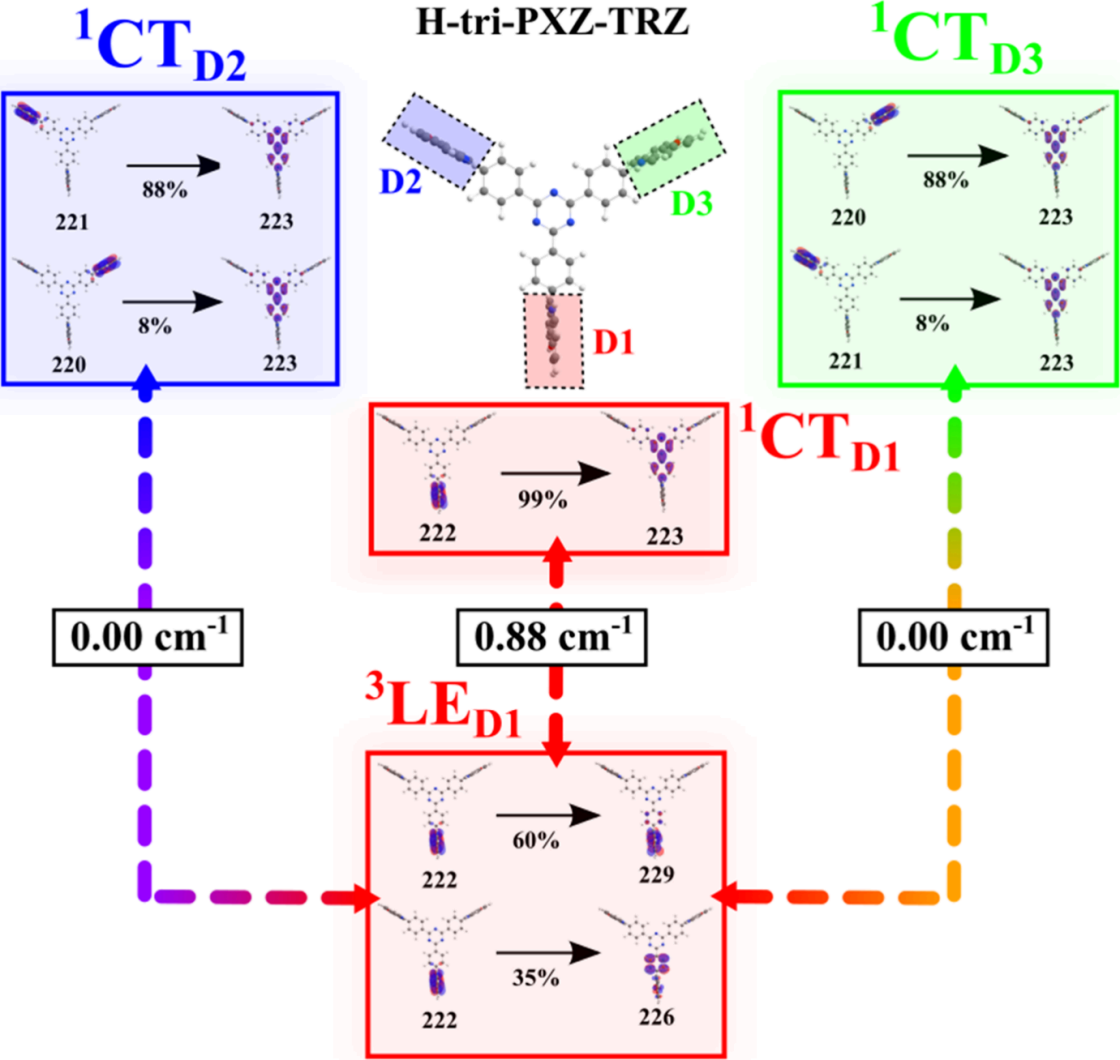
Molecular orbitals involved in the electronic transitions between the ^3^LE_D1_ and respective ^1^CT states.

In terms of transitions involving the ^3^LE_D_ state(s), the presence of three dibromophenoxazine units affords nine potentially possible rISC pathways (3 × ^3^LE_D*i*_)–(3 × ^1^CT_D*j*_), where *i* and *j* = 1–3 indicate the respective active donor units. Interestingly, the molecular orbital analysis and SOC calculations of all possible transitions in both emitters revealed that, among the nine combinations of the ^3^LE_D_ →^1^CT transitions, most of them are, in fact, forbidden. Namely, in the **H-tri-PXZ-TRZ** emitter, the highest SOC constant value (SOC = 0.88 cm^–1^) was observed between the ^3^LE_D_ (^3^LE_D1_) and the ^1^CT (^1^CT_D1_) states which are formed as a result of the electronic density redistribution involving the same orbital, 222 HOMO, or, in other words, involving the same phenoxazine donor D1, as depicted in Figure 8. In contrast to this, ^3^LE_D1_ has no coupling (SOC = 0.00 cm^–1^) with the ^1^CT_D2_ and ^1^CT_D3_ states, which are formed via CT from different donor fragments D2 and D3, respectively, and lack any contribution of the 222 orbital. Therefore, the ^3^LE_D1_ → ^1^CT_D2_ and ^3^LE_D1_ → ^1^CT_D3_ transitions are strictly forbidden.

Another ^3^LE_D_ state of 0.24 eV higher energy is localized on D2 (^3^LE_D2_) and is formed by the orbital 221. Because ^1^CT_D1_ does not involve the 221 orbital, SOC between ^3^LE_D2_ and ^1^CT_D1_ equals zero, as depicted in Figure S12. A stronger coupling of 0.41 cm^–1^ was, however, observed between the ^3^LE_D2_ and ^1^CT_D2_ states thanks to the 88% contribution of the 221 orbital in the formation of the latter state. A much weaker coupling of 0.16 cm^–1^ between the ^3^LE_D2_ and ^1^CT_D3_ states was noticed due to the much lower contribution (8%) of the 221 orbital and thus D2 donor involvement in the formation of the ^1^CT_D3_. Analogously, the ^3^LE_D_ state localized on donor D3 (^3^LE_D3_) formed by the orbital 220 has no coupling (SOC = 0.00 cm^–1^) with the ^1^CT_D1_ state due the lack of shared orbitals. There is a weak coupling (SOC = 0.16 cm^–1^) between ^3^LE_D3_ and ^1^CT_D2_ thanks to the minor contribution (8%) of the 220 orbital in ^1^CT_D2_. A stronger SOC of 0.41 cm^–1^ was observed between ^3^LE_D3_ and ^1^CT_D3_, where the 220 orbital mostly (88%) contributes in ^1^CT_D3_.

Very similar observations were made in the case of **Br-tri-PXZ-TRZ** (Figure S13). The SOC values for the transitions between the ^3^LE_D_ and ^1^CT states are proportional to the percentage of shared HOMO or HOMO–n orbital. The above-mentioned maximal SOC value of 2.67 cm^–1^ is achieved when more than 95% of the donor orbitals are shared, while zero SOC is observed in the case when completely different donors are involved in the ^3^LE_D_ and ^1^CT states’ formation.

From the above-mentioned dependencies, an important conclusion can be drawn. The multiplication of donor fragments in one emitter proportionally increases the density of states and potential number of pathways for rISC. However, to achieve fast and effective ^3^LE–^1^CT transitions with high SOC, it is crucial to ensure significant overlap between the orbitals that form such excited states. The examples given above evidence that different nature and large changes of the orbital momentum do not necessarily guarantee a strong SOC between the ^3^LE and ^1^CT states if they involve electronic density located on different structural fragments. Importantly, the ^3^LE–^1^CT transition involves CT from the neutral ^3^LE state to the zwitterionic ^1^CT one. There is thus an additional condition, namely, the vicinity of the donor and acceptor fragments, so that different transitions can share one of the molecular orbitals and thus provide a pathway for such a CT spin-flip transition.

On the one hand, regarding rISC enhancement via the formation of several rISC pathways, a high degeneracy of pure ^1,3^CT states within the **tri-PXZ-TRZ** scaffold is useless. On the other hand, pure local and CT nature of the ^3^LE_D1_ and ^1^CT_D1_ states, respectively, provide a high SOC constant and thus afford fast microsecond TADF in **H-tri-PXZ-TRZ** and a HA accelerated submicrosecond one in **Br-tri-PXZ-TRZ** in a low-polarity medium. Therefore, among nine possible channels, only one is actually active with SOC = 0.88 and 2.67 cm^–1^, respectively. Our observations are consistent with other recently reported investigations on TADF emitters with similar architecture with *C*_3_ symmetry.^43^ On the other hand, calculations concern the isolated emitter molecule. Two other “inactive” brominated donor fragments as well as brominated donors of neighboring emitters can contribute to the external HA effect especially at high concentrations of **Br-tri-PXZ-TRZ**.

Historically, one of the first three-state TADF models and its more advanced versions were developed to explain the photophysics of very strong D–A- and D–A–D-type TADF emitters, for example, **PTZ-DBTO2**(44) and **diPTZ-DBTO2**.^45^ We can thus answer the above-mentioned question in the following manner: *In contrast to weaker DA-type TADF emitters, in strong ones, theΔE_^1^CT–^3^CT_itself as well as its fluctuations is to low be a critical rISC parameter. It is the negligible SOC value that is the main factor limiting the efficiency of the*^*3*^*CT →*^*1*^*CT transition. Even a minor contribution of the*^*3*^*LE state is thus crucial to enable fast rISC under the condition that the formation of*^*3*^*LE and*^*1*^*CT involves the same structural fragment, donor, or acceptor.*

The results presented here support previously developed TADF models and complete them with the information on the case of (1) strongly stabilized and degenerate ^1,3^CT states with reduced Δ*E*_^1^CT–^3^CT_ and *θ*_DA_ rotation, (2) strong ^3^LE-state impact on rISC, and (3) efficient rISC acceleration by bromine HAs via the ^3^LE–^1^CT SOC enhancement.

## Conclusions

Thanks to the introduction of six bromine atoms into the **H-tri-PXZ-TRZ** structure, the derivative **Br-tri-PXZ-TRZ** reported here exhibits excellent TADF properties. Most importantly, a strong reduction of τ_DF_ together with a significant acceleration of rISC in **Br-tri-PXZ-TRZ** (*k*_rISC_ = 1.75 × 10^6^ s^–1^) was observed compared to that in **H-tri-PXZ-TRZ** (*k*_rISC_ = 0.22 × 10^6^ s^–1^), measured in a popular host material, CBP. What is more, compared to **H-tri-PXZ-TRZ**, **Br-tri-PXZ-TRZ** does not show any notable decrease of PLQY, which makes it a very attractive emitter for red/near-IR OLEDs.

Moreover, double-dopant “hyperfluorescent” systems were tested using **H-tri-PXZ-TRZ** or **Br-tri-PXZ-TRZ** as an assistant dopant combined with the DBP molecule as a terminal emitter in CBP host films. The highest PLQY values of 95% and 86% were observed for the concentration of 1% DBP emitter and 8% **H-tri-PXZ-TRZ** and **Br-tri-PXZ-TRZ**, respectively. Due to efficient FRET, total emission in the optimized hyperfluorescent systems originated from DBP (maximum wavelength *λ*_max_ = 610 nm) with *τ*_DF_ = 2.9 and 1.6 μs, respectively. The best emitter composition for the OLED device is based on 3% DBP and 30% TADF emitter, which differs from the previously reported one.^18^**Br-tri-PXZ-TRZ** shows significantly improved stability, luminance efficiency, and more than 2 times decreased roll-off.

From the DFT/TD-DFT calculations, the following conclusions were drawn:

(1) In both emitters, **H-tri-PXZ-TRZ** and **Br-tri-PXZ-TRZ**, rISC is mediated by the ^3^LE_D_ state thanks to the high SOC *V*_^3^LE→^1^CT_ and energetic closeness of the ^3^LE_D_ and ^1^CT states.

(2) Enhancement of the SOC between the ^3^LE_D_ and ^1^CT states in **Br-tri-PXZ-TRZ** involved in the ^3^LE_D_ → ^1^CT transition is the main reason for accelerated rISC, as observed in time-resolved PL investigations. The introduction of bromine atoms increases the SOC constant *V*_^3^LE→^1^CT_ from 0.88 to 2.67 cm^–1^.

(3) Regarding fast rISC, the increase of the density of states via multiplication of the donor fragment is not helpful in strong DA systems with degenerate CT states. Low SOC values are observed not only for various ^3^CT–^1^CT transitions but also in the case of the ^3^LE_D_ → ^1^CT transitions, where the LE and CT states involve different donor fragments. There is thus additional criteria for the increased SOC of the ^3^LE_D_ → ^1^CT transitions; namely, the states of different nature should share a molecular orbital or a structural fragment to afford effective change of the orbital momentum and increased SOC.

Theoretically calculated rate constants *k*_^3^LE→^1^CT_ perfectly correlate with the experimental *k*_rISC_ and indicate a negligible role of the ^3^CT → ^1^CT transition in such a strong D–A system due to the limited rotational freedom and thus weak vibronic activation of SOC.

## Supporting Information Available

Synthetic procedures and results of analyses, description of the methods used and procedure for determination of the photophysical parameters, computational details and theoretical prediction of the rISC rate constants within *θ*-rotamer models, and solution-processed OLED characterization (PDF)
