# Supplementary material for: Application of the Heavy-Atom Effect for (Sub)microsecond Thermally Activated Delayed Fluorescence and an All-Organic Light-Emitting Device with Low-Efficiency Roll-off
Source: ACS Appl Mater Interfaces. 2024 Mar 18;16(12):15107–20. doi: 10.1021/acsami.3c19627 (PMC10982931; doi:10.1021/acsami.3c19627)
Supplement: Supplementary file 1 — am3c19627_si_001.pdf [file am3c19627_si_001.pdf]

Supporting Information

Application of heavy-atom effect for (sub)microsecond thermally activated delayed fluorescence and all-organic light emitting device with low efficiency roll-off

Michał Mońka,[1]* Szymon Gogoc,[2] Karol Kozakiewicz,[1,3] Vladyslav Ievtukhov,[1,3] Daria Grzywacz,[1,3] Olga Ciupak,[4] Aleksander Kubicki,[1] Piotr Bojarski,[1] Przemyslaw Data,[5]* Illia E. Serdiuk[1]*

[1] Faculty of Mathematics, Physics and Informatics, University of Gdańsk, Wita Stwosza 57, 80-308 Gdańsk, Poland

[2] Faculty of Materials Science and Ceramics, AGH University of Krakow, Mickiewicza 30, 30-059 Krakow, Poland

[3] Faculty of Chemistry, University of Gdansk, Wita Stwosza 63, 80-308 Gdańsk, Poland

[4] Department of Organic Chemistry, Gdańsk University of Technology, Gabriela Narutowicza 11/12, 80-233 Gdańsk, Poland

[5] Faculty of Chemistry, Department of Molecular Physics, Lodz University of Technology, Zeromskiego 116, 90-543 Lodz, Poland

*Corresponding authors. E-mail: illia.serdiuk@ug.edu.pl, michal.monka@ug.edu.pl, przemyslaw.data@p.lodz.pl (OLED), phone + 48 58 523 22 44

TABLE OF CONTENTS

Section S1. Photophysical measurements

Section S2. OLEDs

Section S3. Quantum chemical calculations

Section S4. NMR and MALDI-TOF spectra of Br-tri-PXZ-TRZ

Section S1. Photophysical measurements

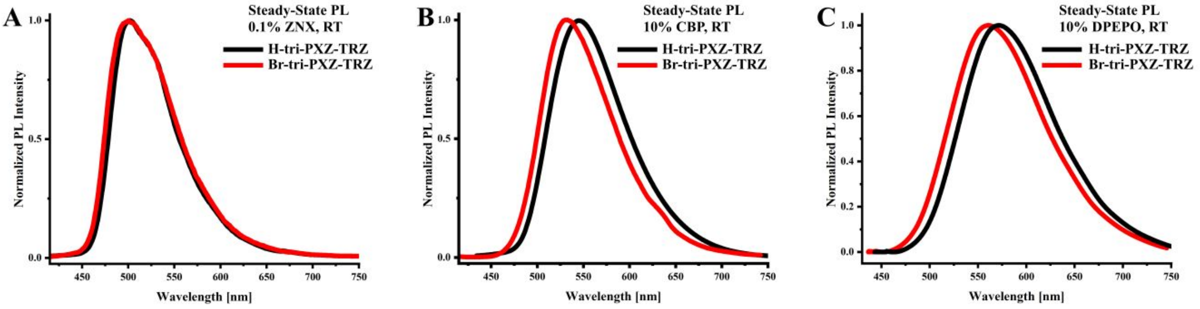

Figure S1. Steady-State PL spectra of **H-tri-PXZ-TRZ** and **Br-tri-PXZ-TRZ** measured in *w/w* 0.1% ZNX (**A**), 10% CBP (**B**) and 10% DPEPO (**C**) hosts at RT, using excitation wavelength λ_{exc} = 330 nm.

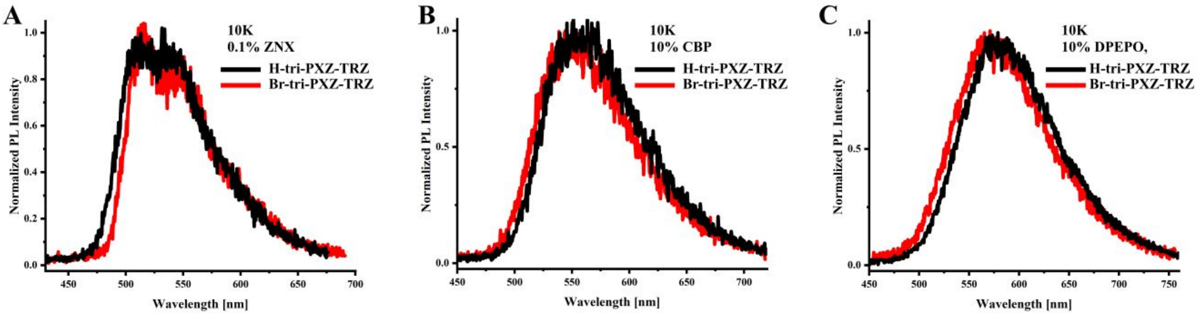

Figure S2. Phosphorescence spectra of investigated emitters measured in 0.1% ZNX (**A**), 10% CBP (**B**) and 10% DPEPO (**C**) at 10K using excitation wavelength λ_{exc} = 330 nm.

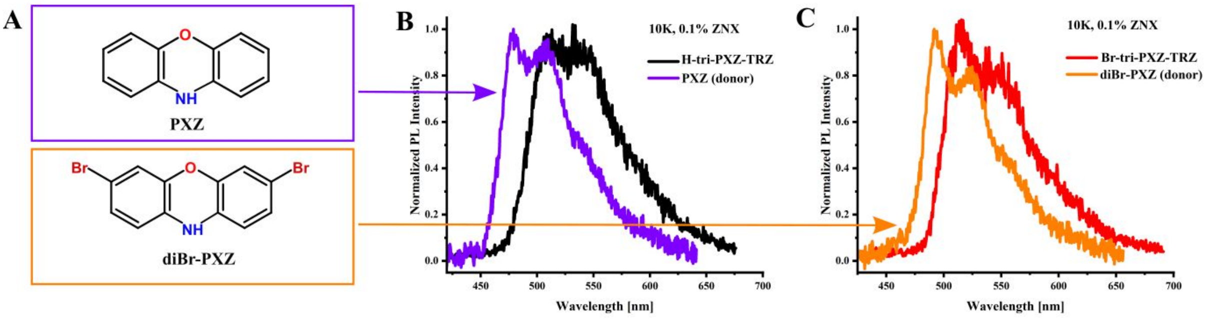

Figure S3. Structures of isolated **PXZ** and **diBr-PXZ** donor fragments **(A)**, comparison of phosphorescence spectra of **H-tri-PXZ-TRZ** with **PXZ** (**B**) and **Br-tri-PXZ-TRZ** with **diBr-PXZ** in 0.1% ZNX at 10K (**C**) using excitation wavelength λ_{exc} = 330 nm.

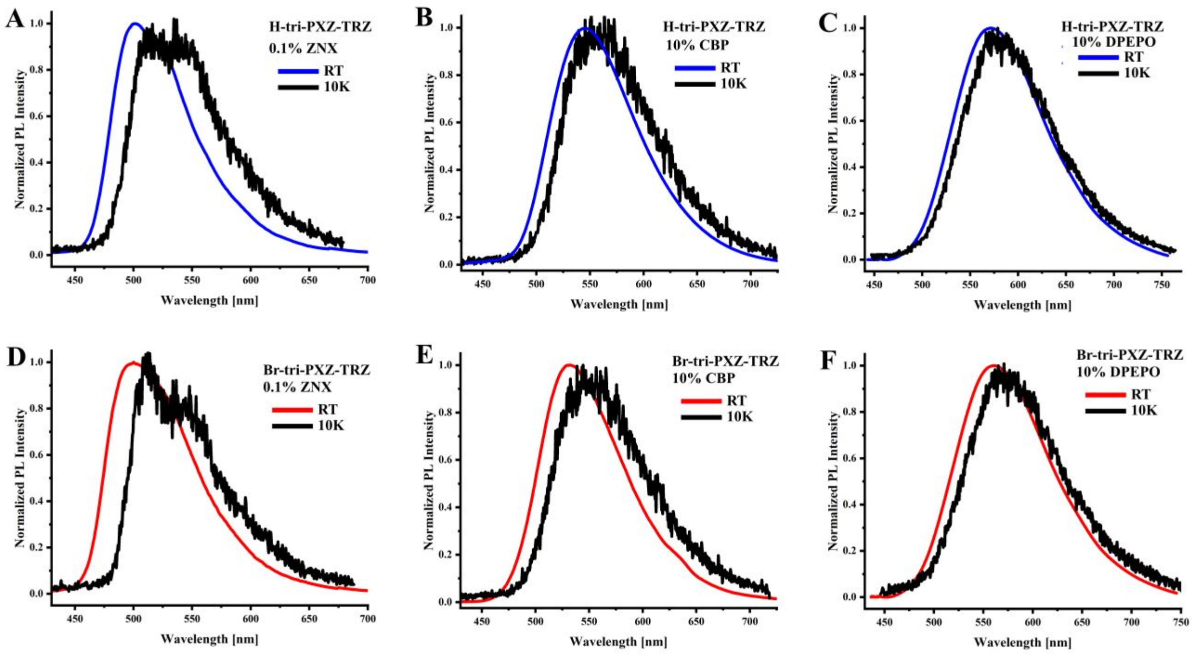

Figure S4. Steady-State PL and phosphoprescence spectra of **H-tri-PXZ-TRZ** (**A-C**) and **Br-tri-PXZ-TRZ** (**D-F**) recorded in ZNX, CBP and DPEPO. Phosphorescence was measured at 10K. All measurements were taken using excitation wavelength λ_{exc} = 330 nm.

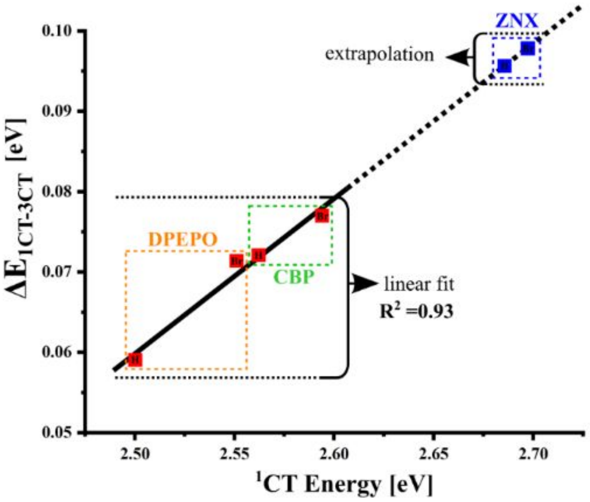

Figure S5. Extrapolation of $\Delta E_{1CT-3CT}$ values as a function of ^{1}CT energy.

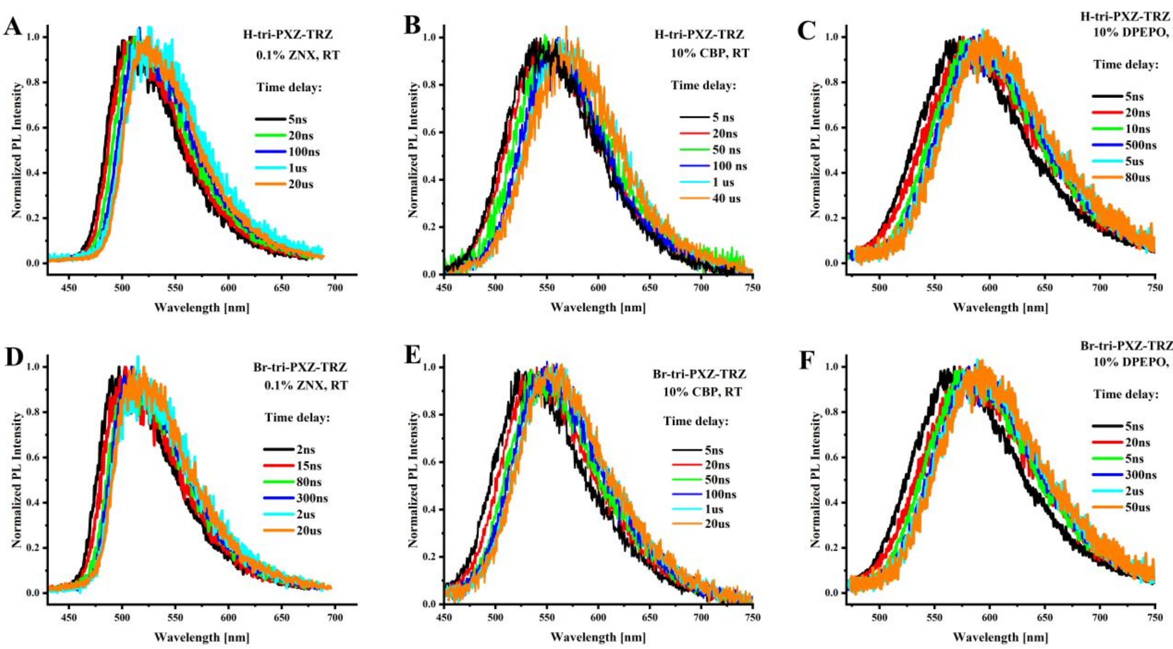

Figure S6. Time-resolved emission spectra (TRES) of **H-tri-PXZ-TRZ** in 0.1% ZNX, (**A**), 10% CBP (**B**), 10% DPEPO (**C**), and Br-tri-PXZ-TRZ in 0.1% ZNX (**D**), 10% CBP (**E**) and 10% DPEPO (**F**). Measurements were taken at room temperature (RT) under vacuum, using excitation wavelength λ_{exc} = 330 nm.

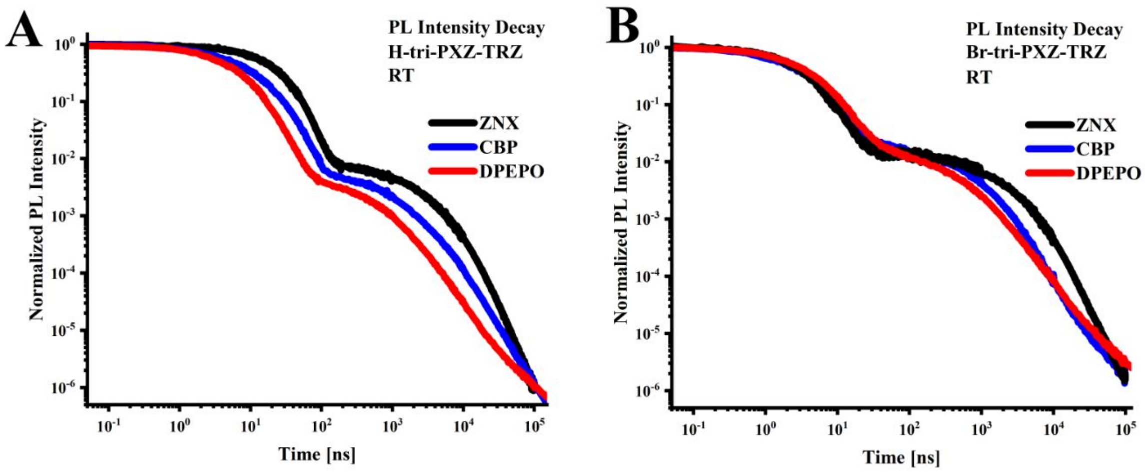

Figure S7. Photoluminescence (PL) intensity decays of **H-tri-PXZ-TRZ** (**A**) and **Br-tri-PXZ-TRZ** (**B**). Measurements were taken at room temperature (RT) under vacuum, using excitation wavelength λ_{exc} = 330 nm.

Determination of photophysical parameters

PL decay curves showed in **Figure 3** (main text) and **Figure S7** were fitted with the multiexponential equation:

$$I(t) = A_0 + \sum_{i=1}^{n} A_i \exp\left(-{}^{t}/_{\tau_i}\right), \tag{S1}$$

where A_i is the pre-exponential factor, τ_i is the decay time and $I(t)$ is emission intensity. Average lifetimes of prompt (τ_{PF}) and delayed fluorescence (τ_{DF}) were determined using formula:

$$\tau_{PF,\ DF} = \sum_{i=1}^{n} f_i \tau_i, \tag{S2}$$

where f_i is fractional contribution of i-th component expressed as:

$$f_i = \frac{A_i \tau_i}{\sum_{i=1}^{n} A_i \tau_i}. \tag{S3}$$

Due to complex function of τ_{PF} and τ_{DF}, the uncertainties $\Delta\tau_{PF}$ and $\Delta\tau_{DF}$ of were estimated using error propagation rule by total differential method[1]:

$$\Delta\tau_{PF,\ DF} = \left|\sum_{i}^{n} \frac{\partial}{\partial f_i}(\tau_{PF,\ DF})\right| \cdot \Delta f_i + \left|\sum_{i}^{n} \frac{\partial}{\partial \tau_i}(\tau_{PF,\ DF})\right| \cdot \Delta\tau_i \tag{S4}$$

where Δf represents an error of the fractional contribution of i-th component:

$$\Delta f_i = \left|\frac{\partial}{\partial A_i}(f_i)\right| \cdot \Delta A_i + \left|\frac{\partial}{\partial \tau_i}(f_i)\right| \cdot \Delta\tau_i \tag{S5}$$

$$\Delta f_i = \left|\frac{\tau_i \sum_j^{n-1}(A_j \tau_j)}{\left(\sum_i^n A_i \tau_i\right)^2}\right| \cdot \Delta A_i + \left|\frac{A_i \sum_j^{n-1}(A_j \tau_j)}{\left(\sum_i^n A_i \tau_i\right)^2}\right| \cdot \Delta\tau_i, j \neq i \tag{S6}$$

and ΔA_i represents an error of i-th amplitude, $\Delta\tau_i$ is decay time error. Both values are determined directly from the multiexponential fitting procedure, based on the least squares method (**Table S1** and **S2**).

The ratio of DF and PF quantum yields $\varphi_{DF}/\varphi_{PF}$ was determined as following:

$$\frac{\varphi_{DF}}{\varphi_{PF}} = \frac{A_{DF}\tau_{DF}}{A_{PF}\tau_{PF}}, \tag{S7}$$

where A_{DF} and A_{PF} are pre-exponential factors of delayed and prompt fluorescence, respectively. Rate constants of radiative (k_r) and nonradiative (k_{nr}) decay and intersystem crossing (k_{ISC}) were given by equations[2]:

$$k_r = \frac{\varphi_{PF}}{\tau_{PF}}, \tag{S8}$$

$$k_{ISC} = \frac{\varphi_{DF}}{\varphi\tau_{PF}}, \tag{S9}$$

$$k_{nr} = \frac{1}{\tau_{PF}} - (k_r + k_{ISC}), \tag{S10}$$

where φ is PLQY ($\varphi_{DF} + \varphi_{PF}$). Further, the quantum yields for ISC and rISC were calculated as

$$\varphi_{ISC} = k_{ISC}\tau_{PF}, \tag{S11}$$

$$\varphi_{rISC} = \frac{1 - \varphi_{PF}/\varphi}{\varphi_{ISC}}. \tag{S12}$$

Finally, rate constant for rISC (k_{rISC}) was calculated as

$$k_{rISC} = \frac{\varphi_{rISC}}{\tau_{DF}}\left(\frac{\varphi}{\varphi_{PF}}\right). \tag{S13}$$

Photophysical parameters are presented in **Table 2** (main text).

Table S1. The parameters of multiexponential fitting procedure in PF region.

ZNX												
	1		2		3		4		5		6	
**H-tri-PXZ-TRZ**	A_1	0.63±0.04	A_2	0.37±0.03	A_3	-----	A_4	-----	A_5	-----	A_6	-----
	τ_1^a	17.41±0.11	τ_2^a	21.74±0.72	τ_3	-----	τ_4	-----	τ_5	-----	τ_6	-----
	f_1^b	0.58±0.02	f_2^b	0.42±0.01	f_3	-----	f_4	-----	f_5	-----	f_6	-----
	**τ_{PF} = 19.2±0.9 ns**											
	1		2		3		4		5		6	
**Br-tri-PXZ-TRZ**	A_1	0.61±0.03	A_2	0.40±0.03	A_3	-----	A_4	-----	A_5	-----	A_6	-----
	τ_1^a	1.92±0.09	τ_2^a	5.92±0.21	τ_3	-----	τ_4	-----	τ_5	-----	τ_6	-----
	f_1^b	0.33±0.02	f_2^b	0.67±0.03	f_3	-----	f_4	-----	f_5	-----	f_6	-----
	**τ_{PF} = 4.7±0.3 ns**											
CBP												
	1		2		3		4		5		6	
**H-tri-PXZ-TRZ**	A_1	0.34±0.03	A_2	0.31±0.04	A_3	0.18±0.02	A_4	-----	A_5	-----	A_6	-----
	τ_1^a	0.98±0.08	τ_2^a	8.08±0.14	τ_3^a	22.01±0.71	τ_4	-----	τ_5	-----	τ_6	-----
	f_1^b	0.05±0.02	f_2^b	0.40±0.01	f_3^b	0.55±0.71	f_4	-----	f_5	-----	f_6	-----
	**τ_{PF} = 15.3±0.7 ns**											
	1		2		3		4		5		6	
**Br-tri-PXZ-TRZ**	A_1	0.45±0.04	A_2	0.41±0.03	A_3	0.13±0.03	A_4	-----	A_5	-----	A_6	-----
	τ_1^a	0.62±0.03	τ_2^a	3.41±0.14	τ_3^a	9.21±0.23	τ_4	-----	τ_5	-----	τ_6	-----
	f_1^b	0.10±0.01	f_2^b	0.48±0.03	f_3^b	0.42±0.05	f_4	-----	f_5	-----	f_6	-----
	**τ_{PF} = 5.5±0.5 ns**											
DPEPO												
	1		2		3		4		5		6	
**H-tri-PXZ-TRZ**	A_1	0.57±0.03	A_2	0.31±0.02	A_3	0.03±0.01	A_4	-----	A_5	-----	A_6	-----
	τ_1^a	2.49±0.14	τ_2^a	13.04±0.24	τ_3^a	35.13±0.54	τ_4	-----	τ_5	-----	τ_6	-----
	f_1^b	0.22±0.04	f_2^b	0.62±0.03	f_3^b	0.16±0.05	f_4	-----	f_5	-----	f_6	-----
	**τ_{PF} = 14.2±0.8 ns**											
	1		2		3		4		5		6	
**Br-tri-PXZ-TRZ**	A_1	0.41±0.05	A_2	0.59±0.02	A_3	-----	A_4	-----	A_5	-----	A_6	-----
	τ_1^a	0.92±0.06	τ_2^a	6.54±0.13	τ_3^a	-----	τ_4	-----	τ_5	-----	τ_6	-----
	f_1^b	0.11±0.02	f_2^b	0.89±0.02	f_3^b	-----	f_4	-----	f_5	-----	f_6	-----
	**τ_{PF} = 6.0±0.6 ns**											

^{a} – individual lifetime of *i*-th component [ns];
^{b} – fractional contribution of *i*-th component [%].

Table S2. The parameters of multiexponential fitting procedure in DF region.

**ZNX**												
	1		2		3		4		5		6	
**H-tri-PXZ-TRZ**	A_1	$[10^{-3}]$ x 5.92±0.03	A_2	$[10^{-3}]$ x 1.65±0.07	A_3	$[10^{-4}]$ x 3.48±0.08	A_4	$[10^{-5}]$ x 2.87±0.12	A_5	-----	A_6	-----
	τ_1^a	1.62±0.13	τ_2^a	5.43±0.44	τ_3^a	9.76±0.23	τ_4^a	24.34±0.29	τ_5^a	-----	τ_6	-----
	f_1	0.43±0.02	f_2	0.39±0.02	f_3	0.15±0.04	f_4	0.03±0.02	f_5	-----	f_6	-----
	**τ_{DF} = 5.0±0.3 us**											
	1		2		3		4		5		6	
**Br-tri-PXZ-TRZ**	A_1	$[10^{-3}]$ x 8.13±0.11	A_2	$[10^{-3}]$ x 3.84±0.04	A_3	$[10^{-4}]$ x 3.47±0.08	A_4	$[10^{-5}]$ x 3.55±0.12	A_5	-----	A_6	-----
	τ_1^a	1.12±0.05	τ_2^a	3.76±0.32	τ_3^a	9.42±0.25	τ_4^a	16.21±0.28	τ_5^a	-----	τ_6	-----
	f_1	0.32±0.02	f_2	0.55±0.06	f_3	0.12±0.03	f_4	0.02±0.01	f_5	-----	f_6	-----
	**τ_{DF} = 3.9±0.4 us**											
**CBP**												
	1		2		3		4		5		6	
**H-tri-PXZ-TRZ**	A_1	$[10^{-3}]$ x 1.45±0.12	A_2	$[10^{-3}]$ x 1.44±0.17	A_3	$[10^{-4}]$ x 2.73±0.04	A_4	$[10^{-5}]$ x 3.77±0.28	A_5	$[10^{-6}]$ x 2.75±0.22	A_6	$[10^{-7}]$ x 2.25±0.42
	τ_1^a	0.41±0.04	τ_2^a	1.42±0.08	τ_3^a	4.85±0.32	τ_4^a	14.12±0.32	τ_5^a	50.61±0.92	τ_6^a	247.61±3.21
	f_1	0.12±0.02	f_2	0.43±0.05	f_3	0.31±0.09	f_4	0.11±0.03	f_5	0.03±0.03	f_6	0.01±0.05
	**τ_{DF} = 8.2±0.5 ns**											
	1		2		3		4		5		6	
**Br-tri-PXZ-TRZ**	A_1	$[10^{-3}]$ x 6.05±0.32	A_2	$[10^{-3}]$ x 5.24±0.17	A_3	$[10^{-3}]$ x 2.98±0.04	A_4	$[10^{-3}]$ x 1.07±0.28	A_5	$[10^{-4}]$ x 1.06±0.24	A_6	$[10^{-5}]$ x 1.02±0.22
	τ_1^a	0.11±0.02	τ_2^a	0.52±0.08	τ_3^a	1.15±0.42	τ_4^a	2.33±0.22	τ_5^a	6.33±0.72	τ_6^a	23.34±1.21
	f_1	0.05±0.02	f_2	0.25±0.06	f_3	0.33±0.29	f_4	0.26±0.05	f_5	0.07±0.03	f_6	0.02±0.05
	**τ_{DF} = 2.1±0.3 us**											
**DPEPO**												
	1		2		3		4		5		6	
**H-tri-PXZ-TRZ**	A_1	$[10^{-4}]$ x 4.62±0.38	A_2	$[10^{-4}]$ x 2.01±0.37	A_3	$[10^{-5}]$ x 3.67±0.24	A_4	$[10^{-6}]$ x 4.27±0.48	A_5	$[10^{-7}]$ x 5.76±0.64	A_6	$[10^{-7}]$ x 1.22±0.12
	τ_1^a	0.33±0.06	τ_2^a	0.99±0.11	τ_3^a	3.65±0.42	τ_4^a	11.23±0.22	τ_5^a	33.24±0.72	τ_6^a	238.12±7.21
	f_1	0.21±0.04	f_2	0.45±0.08	f_3	0.19±0.09	f_4	0.07±0.05	f_5	0.03±0.03	f_6	0.05±0.09
	**τ_{PF} = 15.8±0.8 us**											
	1		2		3		4		5		6	
**Br-tri-PXZ-TRZ**	A_1	$[10^{-3}]$ x 3.15±0.33	A_2	$[10^{-3}]$ x 2.31±0.44	A_3	$[10^{-4}]$ x 6.42±0.54	A_4	$[10^{-5}]$ x 8.11±0.76	A_5	$[10^{-6}]$ x 4.56±0.44	A_6	$[10^{-7}]$ x 9.12±0.22
	τ_1^a	0.11±0.03	τ_2^a	0.59±0.14	τ_3^a	1.52±0.23	τ_4^a	4.43±0.54	τ_5^a	15.74±0.53	τ_6^a	118.12±3.24
	f_1	0.11±0.03	f_2	0.43±0.05	f_3	0.30±0.11	f_4	0.11±0.06	f_5	0.02±0.03	f_6	0.03±0.08
	**τ_{PF} = 6.3±0.3 us**											

^{a} – individual lifetime of *i*-th component [ns];

^{b} – fractional contribution of *i*-th component [%].

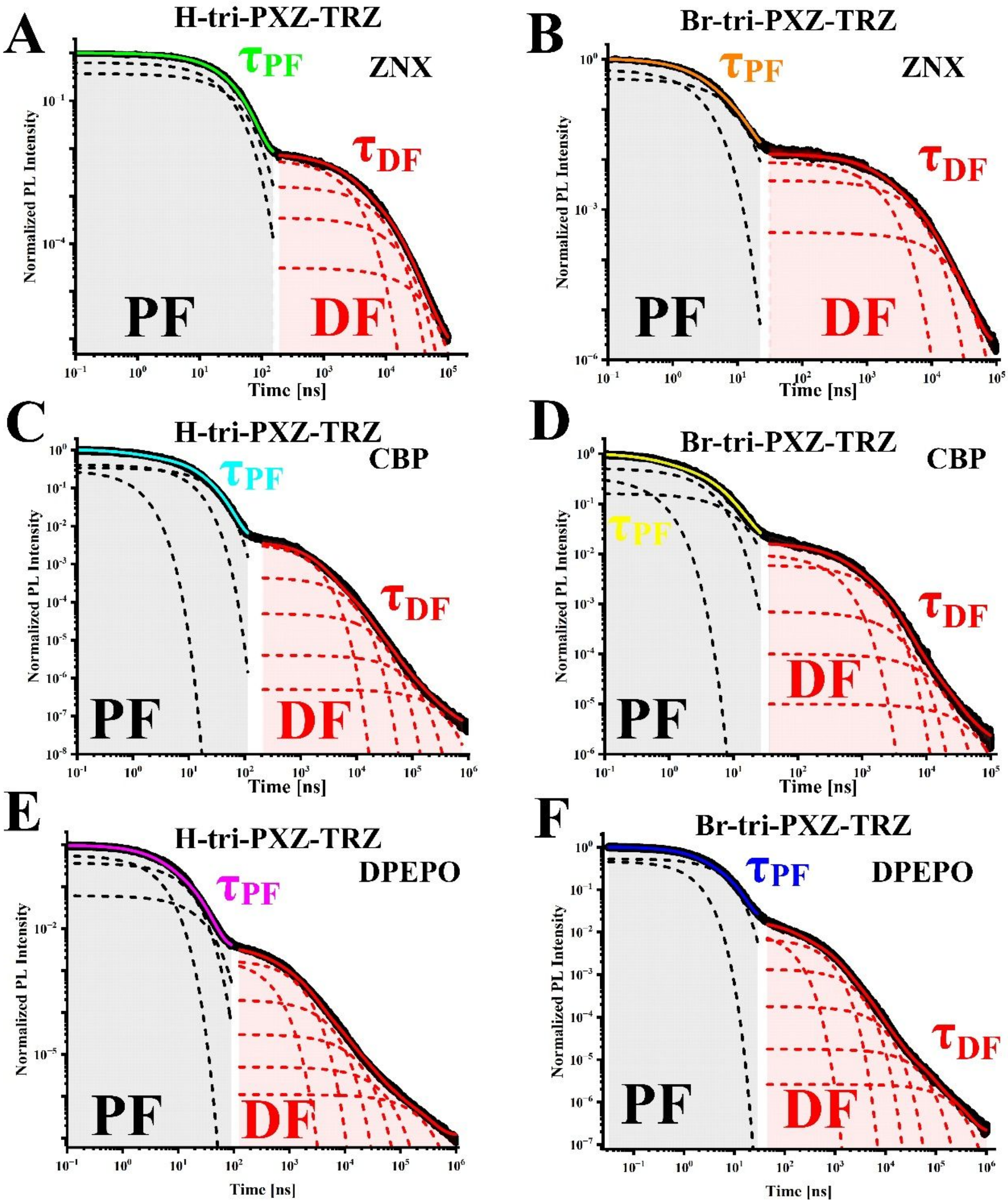

Figure S8. PL intensity decay profiles of **H-tri-PXZ-TRZ** (**A, C, E**) and **Br-tri-PXZ-TRZ** (**B, D, F**) with fitted exponential curves. PF – prompt fluorescence, DF – delayed fluorescence. Measurements were taken at room temperature (RT) under vacuum, using excitation wavelength λ_{exc} = 330 nm.

Section S2. OLEDs

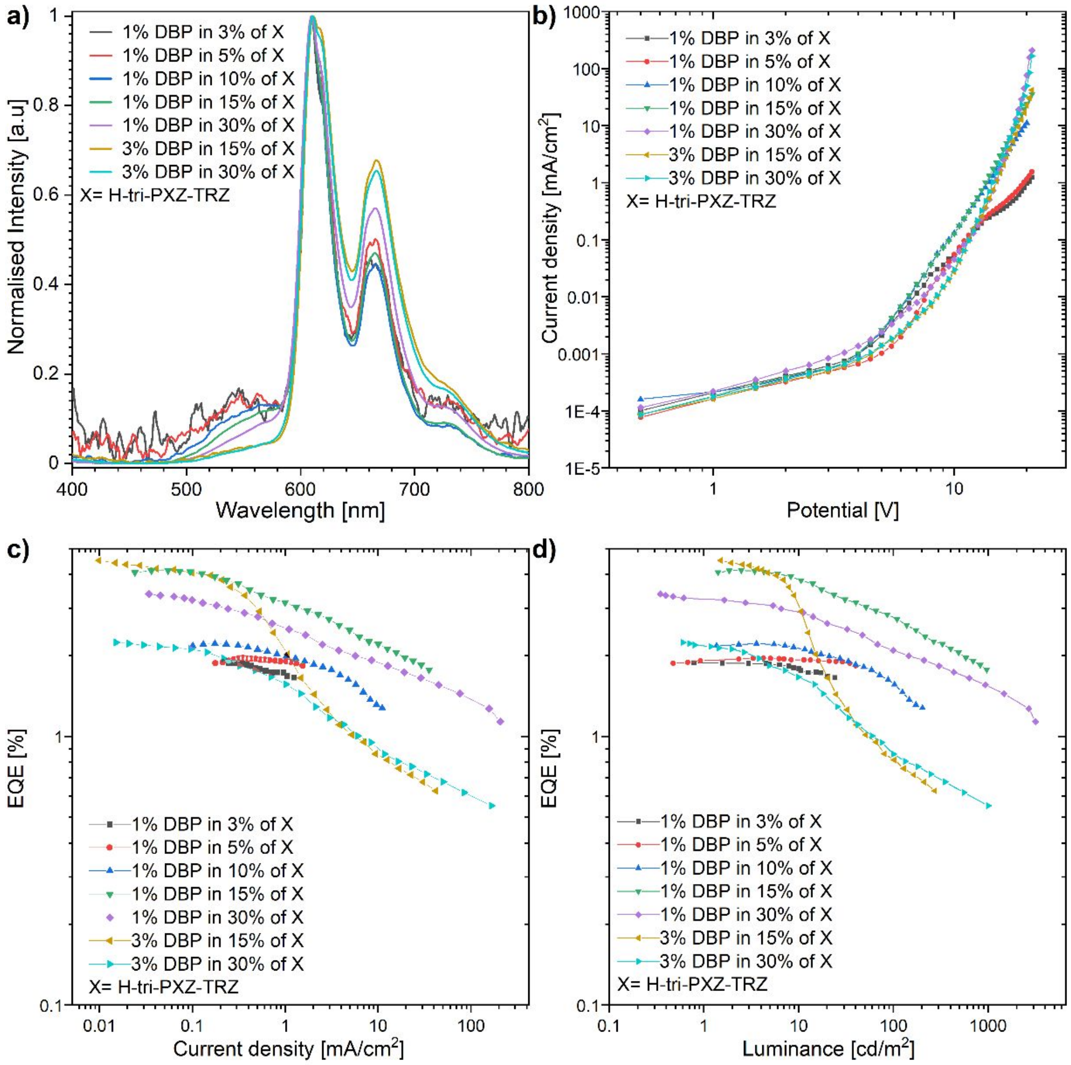

Figure S9. The characteristics of the OLED devices ITO /PEDOT:PSS (40 nm)/ emitter in CBP (30 nm)/ TPBi [2,2',2''-(1,3,5- benzinetriyl)-tris(1-phenyl-1-H-benzimidazole)] (50 nm)/LiF (1 nm)/Al (100 nm)]. Electroluminescence spectra (a). Current density-bias characteristic (b). EQE – current density (c). EQE - luminance characteristics (d).

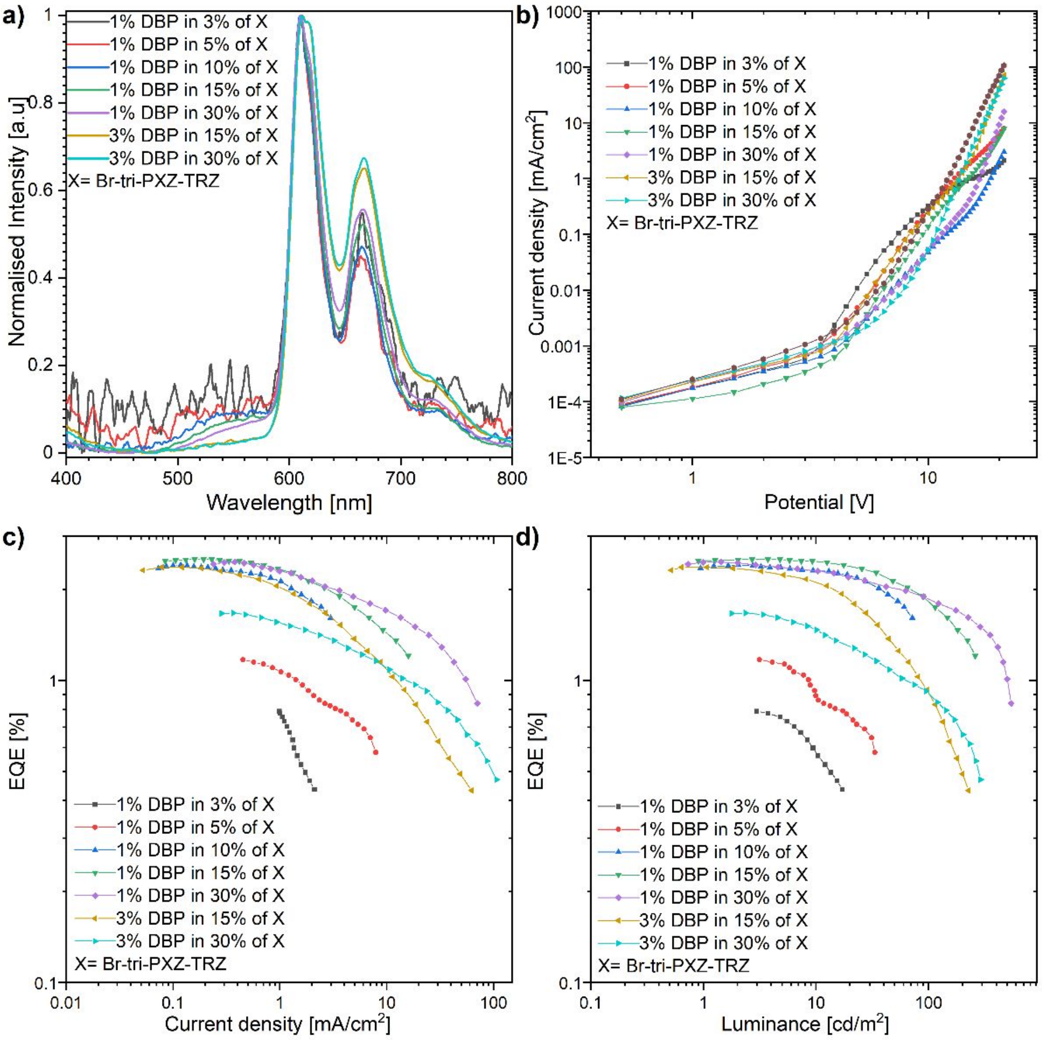

Figure S10. The characteristics of the OLED devices ITO /PEDOT:PSS (40 nm)/ emitter in CBP (30 nm)/ TPBi [2,2',2''-(1,3,5-benzinetriyl)-tris(1-phenyl-1-H-benzimidazole)] (50 nm)/LiF (1 nm)/Al (100 nm)]. Electroluminescence spectra (a). Current density-bias characteristic (b). EQE – current density (c). EQE - luminance characteristics (d).

Section S3. Quantum chemical calculations

Theoretical constant rates of rISC were calculated using Marcus-Hush equation[3]:

$$k_{\mathrm{rISC}} = \frac{V^2}{\hbar} \cdot \sqrt{\frac{\pi}{k_B \mathrm{T} \lambda}} \exp\left(- \frac{(\Delta \mathrm{E_{ST}} + \lambda)^2}{4 k_B \mathrm{T} \lambda} \right), \tag{S14}$$

where:

- k_B – Boltzmann constant,
- λ – reorganization energy,
- V – spin-orbit coupling (SOC) constant,
- T – temperature,
- $\Delta \mathrm{E_{ST}}$ – energy gap between singlet and triplet states.

However, due to different mechanisms of ^{3}CT→^{1}CT and ^{3}LE$_D$→^{1}CT transitions, equation (S14) has to be implemented as follows.

rISC: ^{3}CT→^{1}CT transition (θ_{DA}-rotamers model)

Within the θ_{DA}-rotamers model[4], (^{3}CT→^{1}CT transition) the total constant rate ($k_{3\mathrm{CT}\to1\mathrm{CT}}$) consists of individual rate constant $k_{3\mathrm{CT}\to1\mathrm{CT}}[\theta_{\mathrm{DA}}]$, originating from various molecules with different values of θ_{DA} (as depicted in **Figure 6A**, main text):

$$k_{3\mathrm{CT}\to1\mathrm{CT}} = \sum^{n} p_\theta[\theta_{\mathrm{DA}}] \cdot k_{3\mathrm{CT}\to1\mathrm{CT}}[\theta_{\mathrm{DA}}], \tag{S15}$$

where:

- $p_\theta[\theta_{\mathrm{DA}}]$ = Boltzmann distribution function (population of θ_{DA}-rotamers),
- $k_{3\mathrm{CT}\to1\mathrm{CT}}[\theta_{\mathrm{DA}}]$ – the individual constant rates of specified θ_{DA}-rotamer.

$$p_\theta[\theta_{\mathrm{DA}}] = \frac{\exp\left(\frac{\Delta E_\theta[\theta_{\mathrm{DA}}]}{k_B T}\right)}{\sum^{n} \exp\left(\frac{\Delta E_\theta[\theta_{\mathrm{DA}}]}{k_B T}\right)}, \tag{S16}$$

- $\Delta E_\theta[\theta_{\mathrm{DA}}]$ - the energy difference between specified θ_{DA}-rotamer and optimized structure in ^{3}CT state.

The individual constant rates of rISC within $^3CT \rightarrow ^1CT$ transition ($k_{3CT\rightarrow 1CT}[\theta_{DA}]$) were calculated using Marcus-Hush equation:

$$k_{3CT\rightarrow 1CT}[\theta_{DA}] = \frac{V_{3CT\rightarrow 1CT}[\theta_{DA}]^2}{\hbar} \cdot \sqrt{\frac{\pi}{k_B T \lambda_{3CT\rightarrow 1CT}[\theta_{DA}]}} \exp\left(- \frac{(\Delta E_{3CT\rightarrow 1CT}[\theta_{DA}] + \lambda_{3CT\rightarrow 1CT}[\theta_{DA}])^2}{4 k_B T \lambda_{3CT\rightarrow 1CT}[\theta_{DA}]} \right), \qquad \text{(S17)}$$

Where are 3 parameters:

- $\lambda_{3CT\rightarrow 1CT}[\theta_{DA}]$ – reorganization energy during $^3CT \rightarrow ^1CT$ transition of specified θ_{DA}-rotamer with θ_{DA} value of dihedral angle between D-A,
- $V_{3CT\rightarrow 1CT}[\theta_{DA}]$ – SOC constant between ^3CT-^1CT states of specified θ_{DA}-rotamer with θ_{DA},
- $\Delta E_{3CT\rightarrow 1CT}[\theta_{DA}]$ – energy gap between ^3CT-^1CT states of specified θ_{DA}-rotamer with θ_{DA}.

Table S3. Computational data for rISC constant rate prediction within $^3CT \rightarrow ^1CT$ transition using θ_{DA}-rotamers model for **H-tri-PXZ-TRZ**.

A	θ_{DA} [°]	p_θ [θ_{DA}] [%]	$V_{3CT\rightarrow 1CT}[\theta_{DA}]$ [cm^{-1}]	$\Delta E_{1CT-3CT}[\theta_{DA}]$ [meV]	$\lambda_{3CT\rightarrow 1CT}[\theta_{DA}]$ [meV]	$k_{3CT\rightarrow 1CT}[\theta_{DA}]$ [10^4 s^{-1}]	$p_\theta \cdot k_{3CT\rightarrow 1CT}[\theta_{DA}]$ [10^4 s^{-1}]
**-5.0**	74.7	0.00	0.063	587.35	821.93	0.000	0.0000
**-4.8**	75.3	0.00	0.060	541.37	757.56	0.000	0.0000
**-4.4**	76.5	0.01	0.055	455.05	636.72	0.000	0.0000
**-4.2**	77.1	0.01	0.053	414.71	580.23	0.000	0.0000
**-4.0**	77.7	0.02	0.050	376.24	526.38	0.000	0.0000
**-3.8**	78.4	0.04	0.048	339.65	475.15	0.000	0.0000
**-3.6**	78.9	0.06	0.045	304.93	426.54	0.000	0.0000
**-3.4**	79.6	0.11	0.043	272.09	380.57	0.000	0.0000
**-3.0**	80.1	0.28	0.038	212.04	296.50	0.000	0.0000
**-2.6**	82.1	0.63	0.033	159.50	222.93	0.001	0.0000
**-2.4**	82.8	0.90	0.030	136.04	190.09	0.002	0.0000
**-2.2**	83.3	1.26	0.028	114.46	159.88	0.005	0.0001
**-2.0**	83.9	1.72	0.025	94.75	132.29	0.012	0.0002
**-1.6**	85.1	2.90	0.020	60.97	85.00	0.046	0.0014
**-1.2**	86.3	4.37	0.015	34.69	48.21	0.119	0.0052
**-0.8**	87.5	5.86	0.010	15.92	21.93	0.189	0.0111
**-0.6**	88.2	6.49	0.008	9.35	12.73	0.189	0.0123
**-0.4**	88.7	6.98	0.005	4.66	6.16	0.150	0.0104
**-0.2**	89.4	7.29	0.003	1.84	2.22	0.070	0.0051
**-0.1**	89.7	7.37	0.001	1.14	1.23	0.024	0.0017
**0.0**	90.0	7.40	0.000	0.90	0.90	0.000	0.0000
**0.1**	90.3	7.37	0.001	1.13	1.23	0.024	0.0017
**0.2**	90.6	7.29	0.003	1.84	2.21	0.070	0.0051
**0.4**	91.2	6.98	0.005	4.65	6.15	0.150	0.0105
**0.6**	91.8	6.49	0.008	9.33	12.71	0.189	0.0123
**0.8**	92.4	5.86	0.010	15.90	21.90	0.189	0.0111
**1.2**	93.6	4.37	0.015	34.66	48.16	0.119	0.0052
**1.6**	94.9	2.90	0.020	60.92	84.93	0.047	0.0014
**2.0**	96.1	1.72	0.025	94.69	132.20	0.012	0.0002
**2.2**	96.7	1.26	0.028	114.39	159.78	0.005	0.0001
**2.4**	97.3	0.90	0.030	135.96	189.99	0.002	0.0000
**2.6**	97.9	0.63	0.033	159.42	222.82	0.001	0.0000
**3.0**	99.1	0.28	0.038	211.95	296.37	0.000	0.0000
**3.3**	100.1	0.11	0.043	271.99	380.42	0.000	0.0000
**3.6**	101.0	0.06	0.045	304.82	426.39	0.000	0.0000
**3.8**	101.6	0.04	0.048	339.53	474.98	0.000	0.0000
**4.0**	102.2	0.02	0.050	376.12	526.20	0.000	0.0000
**4.2**	102.8	0.01	0.053	414.58	580.05	0.000	0.0000
**4.4**	103.4	0.01	0.055	454.92	636.53	0.000	0.0000
**4.8**	104.7	0.00	0.060	541.23	757.36	0.000	0.0000
**5.0**	105.3	0.00	0.063	587.20	821.72	0.000	0.0000
			Statistical sum (eq. S15): $\Sigma\, p_\theta \cdot k_{3CT\rightarrow 1CT}$ [10^4 s^{-1}]				0.0951

Table S4. Computational data for rISC constant rate prediction within ^{3}CT→^{1}CT transition using θ_{DA}-rotamers model for **Br-tri-PXZ-TRZ**.

A	θ_{DA} [°]	$p_\theta[\theta_{DA}]$ [%]	$V_{3CT\to1CT}[\theta_{DA}]$ [cm^{-1}]	$\Delta E_{1CT-3CT}[\theta_{DA}]$ [meV]	$\lambda_{3CT\to1CT}[\theta_{DA}]$ [meV]	$k_{3CT\to1CT}[\theta_{DA}]$ [10^4 s^{-1}]	$p_\theta \cdot k_{3CT\to1CT}[\theta_{DA}]$ [10^4 s^{-1}]
**-5.0**	74.6	0.00	0.0766	681.48	892.30	0.000	0.0000
**-4.8**	75.5	0.00	0.0735	628.15	822.42	0.000	0.0000
**-4.4**	76.3	0.01	0.0674	528.02	691.22	0.000	0.0000
**-4.2**	77.0	0.02	0.0643	481.22	629.89	0.000	0.0000
**-4.0**	77.6	0.04	0.0613	436.59	571.42	0.000	0.0000
**-3.8**	78.3	0.06	0.0582	394.15	515.80	0.000	0.0000
**-3.6**	78.8	0.10	0.0551	353.88	463.03	0.000	0.0000
**-3.4**	79.6	0.16	0.0521	315.78	413.11	0.000	0.0000
**-3.0**	80.2	0.37	0.0459	246.12	321.83	0.000	0.0000
**-2.6**	82.0	0.77	0.0398	185.17	241.96	0.000	0.0000
**-2.4**	82.7	1.07	0.0368	157.96	206.31	0.001	0.0000
**-2.2**	83.4	1.45	0.0337	132.92	173.50	0.003	0.0000
**-2.0**	83.8	1.91	0.0306	110.07	143.55	0.009	0.0002
**-1.6**	85.2	3.07	0.0245	70.88	92.21	0.043	0.0013
**-1.2**	86.4	4.43	0.0184	40.40	52.27	0.132	0.0059
**-0.8**	87.5	5.77	0.0123	18.63	23.74	0.237	0.0137
**-0.6**	88.1	6.32	0.0092	11.01	13.75	0.248	0.0157
**-0.4**	88.8	6.75	0.0061	5.56	6.61	0.202	0.0137
**-0.2**	89.5	7.03	0.0031	2.29	2.33	0.095	0.0067
**-0.1**	89.8	7.10	0.0015	1.47	1.26	0.032	0.0023
**0.0**	90.0	7.12	0.0000	1.20	0.90	0.000	0.0000
**0.1**	90.2	7.10	0.0015	1.47	1.25	0.032	0.0023
**0.2**	90.5	7.03	0.0031	2.28	2.32	0.096	0.0067
**0.4**	91.3	6.75	0.0061	5.55	6.59	0.203	0.0137
**0.6**	91.8	6.32	0.0092	10.98	13.72	0.249	0.0157
**0.8**	92.4	5.77	0.0123	18.60	23.70	0.238	0.0137
**1.2**	93.8	4.43	0.0184	40.36	52.21	0.132	0.0059
**1.6**	94.9	3.07	0.0245	70.82	92.13	0.043	0.0013
**2.0**	96.1	1.91	0.0306	110.00	143.46	0.009	0.0002
**2.2**	96.6	1.45	0.0337	132.85	173.40	0.003	0.0000
**2.4**	97.3	1.07	0.0368	157.87	206.20	0.001	0.0000
**2.6**	97.8	0.77	0.0398	185.08	241.84	0.000	0.0000
**3.0**	99.2	0.37	0.0459	246.02	321.69	0.000	0.0000
**3.3**	100.3	0.16	0.0521	315.66	412.95	0.000	0.0000
**3.6**	101.0	0.10	0.0551	353.75	462.86	0.000	0.0000
**3.8**	101.3	0.06	0.0582	394.01	515.62	0.000	0.0000
**4.0**	102.4	0.04	0.0613	436.45	571.23	0.000	0.0000
**4.2**	102.8	0.02	0.0643	481.07	629.69	0.000	0.0000
**4.4**	103.4	0.01	0.0674	527.86	691.01	0.000	0.0000
**4.8**	104.8	0.00	0.0735	627.98	822.20	0.000	0.0000
**5.0**	105.4	0.00	0.0766	681.30	892.07	0.000	0.0000
				Statistical sum (eq. S15): $\Sigma\, p_\theta \cdot k_{3CT\to1CT}$		[10^4 s^{-1}]	0.1196

rISC: $^3LE_D \rightarrow ^1CT$ transition

The constant rates of rISC within $^3LE_D \rightarrow ^1CT$ transition ($k_{3LE\rightarrow 1CT}$) were calculated using Marcus-Hush equation:

$$k_{3LE\rightarrow 1CT} = \frac{V_{3LE\rightarrow 1CT}{}^2}{\hbar} \cdot \sqrt{\frac{\pi}{k_B T \lambda_{3LE\rightarrow 1CT}}} \exp\left(-\frac{(\Delta E_{3LE\rightarrow 1CT} + \lambda_{3LE\rightarrow 1CT})^2}{4 k_B T \lambda_{3LE\rightarrow 1CT}}\right), \quad \text{(S18)}$$

Where are 3 parameters:

- $\lambda_{3LE\rightarrow 1CT}$ – reorganization energy during $^3LE_D \rightarrow ^1CT$ transition,
- $V_{3LE\rightarrow 1CT}$ – SOC constant between ^3LE_D -^1CT states,
- $\Delta E_{3LE\rightarrow 1CT}$ – energy gap between ^3LE_D -^1CT states.

According to alignment of triplet excited states depicted in **Figure 2** (main text), in order to calculate constant rates correctly, it is necessary to take into account the population of excited states (χT_1 and χT_2), calculated using Boltzmann distribution. The results are presented in the **Table S5**.

Table S5. Alignment of excited triplet states with calculated population in considered media.

		T_1		T_2		$\Delta E_{T1\text{-}T1}$	$\Delta E_{T1\text{-}T2}$	a_1	a_2	χT_1	χT_2
			[eV]		[eV]	[eV]	[eV]			[%]	[%]
**H**	ZNX	^3LE_D	2.59	^3CT	2.60	0.00	0.01	1.000	0.798	55.7	44.3
**Br**		^3LE_D	2.58	^3CT	2.61	0.00	0.03	1.000	0.501	66.7	33.3
**H**	CBP	^3CT	2.49	^3LE_D	2.59	0.00	0.10	1.000	0.017	98.3	1.7
**Br**		^3CT	2.51	^3LE_D	2.58	0.00	0.07	1.000	0.058	94.5	5.5
**H**	DPEPO	^3CT	2.44	^3LE_D	2.59	0.00	0.15	1.000	0.003	99.7	0.3
**Br**		^3CT	2.48	^3LE_D	2.58	0.00	0.10	1.000	0.015	98.5	1.5

Table S6. Computational data for rISC constant rate prediction within $^3LE_D \rightarrow ^1CT$ transition for **H-** and **Br-tri-PXZ-TRZ** in investigated media.

		$\Delta E_{1CT\text{-}3LE}$	$\lambda_{3LE\rightarrow 1CT}$	$V_{3LE\rightarrow 1CT}$	$k_{3LE\rightarrow 1CT}$	χ_{3LE}	$\chi_{3LE}\, k_{3LE\rightarrow 1CT}$
		[eV]	[eV]	[cm^{-1}]	[10^4 s^{-1}]	[%]	[10^4 s^{-1}]
**H**	ZNX	0.10	0.24	0.88	79.1	55.7	44.1
**Br**		0.12	0.28	2.67	359.7	66.7	240.2
**H**	CBP	-0.04	0.24	0.88	1163.4	1.7	19.8
**Br**		0.01	0.28	2.67	3682.6	5.5	201.8
**H**	DPEPO	-0.09	0.24	0.88	3206.3	0.3	9.2
**Br**		-0.03	0.28	2.67	8207.5	1.5	112.7

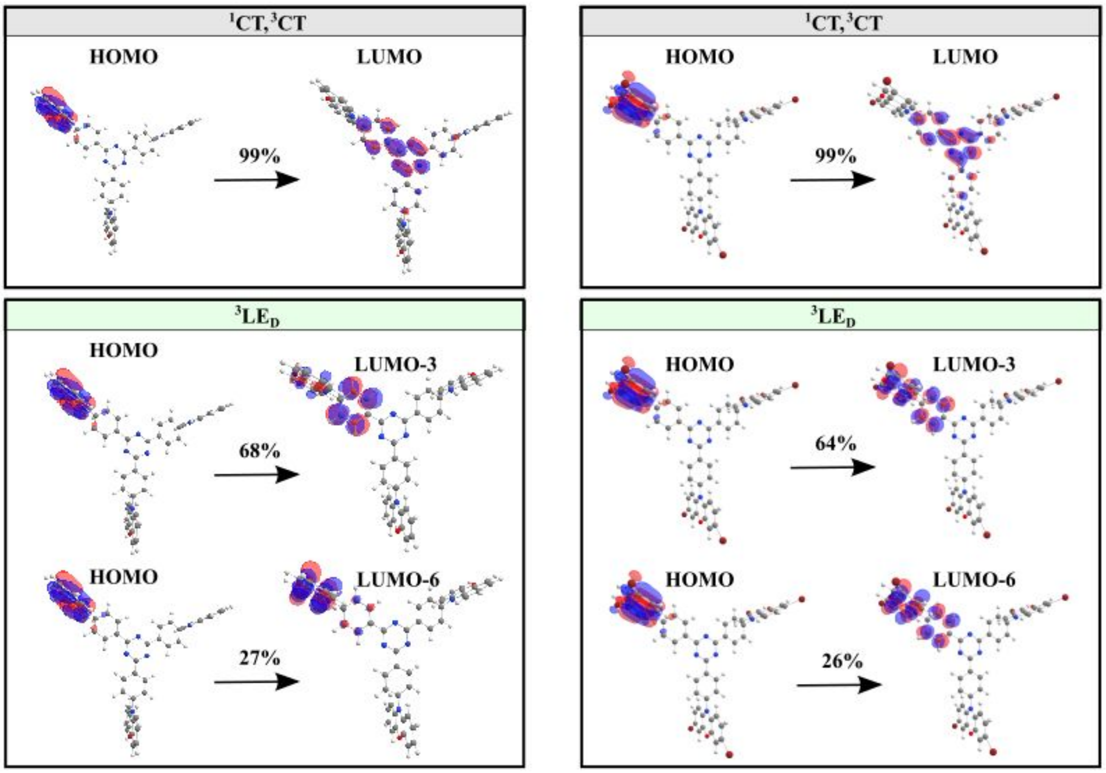

Figure S11. Molecular orbitals involved in the formation of key excited electronic states (left: **H-tri-PXZ-TRZ**, right: **Br-tri-PXZ-TRZ**).

Table S7. SOC constant values between ^{3}CT-^{1}CT states for **H-tri-PXZ-TRZ**.

A	θ_{DA}	$^3CT_1$-$^1CT_1$	$^3CT_1$-$^1CT_2$	$^3CT_1$-$^1CT_3$	$^3CT_2$-$^1CT_1$	$^3CT_2$-$^1CT_2$	$^3CT_2$-$^1CT_3$	$^3CT_3$-$^1CT_1$	$^3CT_3$-$^1CT_2$	$^3CT_3$-$^1CT_3$
	[°]	[cm^{-1}]	[cm^{-1}]	[cm^{-1}]	[cm^{-1}]	[cm^{-1}]	[cm^{-1}]	[cm^{-1}]	[cm^{-1}]	[cm^{-1}]
**-5.0**	74.7	0.063	0.051	0.045	0.082	0.092	0.077	0.059	0.080	0.066
**-4.8**	75.3	0.060	0.049	0.043	0.079	0.088	0.074	0.056	0.077	0.063
**-4.4**	76.5	0.055	0.045	0.040	0.072	0.081	0.068	0.052	0.071	0.058
**-4.2**	77.1	0.053	0.044	0.038	0.069	0.077	0.065	0.050	0.068	0.055
**-4.0**	77.7	0.050	0.042	0.037	0.066	0.074	0.063	0.048	0.065	0.053
**-3.8**	78.4	0.048	0.040	0.035	0.063	0.070	0.060	0.046	0.062	0.050
**-3.6**	78.9	0.045	0.038	0.034	0.060	0.066	0.057	0.044	0.059	0.047
**-3.4**	79.6	0.043	0.036	0.032	0.057	0.062	0.054	0.041	0.056	0.045
**-3.0**	80.1	0.038	0.033	0.029	0.051	0.055	0.048	0.037	0.050	0.040
**-2.6**	82.1	0.033	0.029	0.026	0.045	0.048	0.042	0.033	0.044	0.034
**-2.4**	82.8	0.030	0.027	0.024	0.042	0.044	0.040	0.031	0.041	0.032
**-2.2**	83.3	0.028	0.025	0.023	0.039	0.040	0.037	0.029	0.038	0.029
**-2.0**	83.9	0.025	0.023	0.021	0.036	0.037	0.034	0.026	0.035	0.026
**-1.6**	85.1	0.020	0.020	0.018	0.030	0.029	0.028	0.022	0.029	0.021
**-1.2**	86.3	0.015	0.016	0.015	0.023	0.022	0.022	0.018	0.023	0.016
**-0.8**	87.5	0.010	0.012	0.011	0.017	0.015	0.017	0.014	0.017	0.011
**-0.6**	88.2	0.008	0.011	0.010	0.014	0.011	0.014	0.011	0.014	0.008
**-0.4**	88.7	0.005	0.009	0.008	0.011	0.007	0.011	0.009	0.011	0.005
**-0.2**	89.4	0.003	0.007	0.007	0.008	0.004	0.008	0.007	0.008	0.003
**-0.1**	89.7	0.001	0.006	0.006	0.007	0.002	0.006	0.006	0.007	0.001
**0.0**	90.0	0.000	0.005	0.005	0.005	0.000	0.005	0.005	0.005	0.000
**0.1**	90.3	0.001	0.006	0.006	0.007	0.002	0.006	0.006	0.007	0.001
**0.2**	90.6	0.003	0.007	0.007	0.008	0.004	0.008	0.007	0.008	0.003
**0.4**	91.2	0.005	0.009	0.008	0.011	0.007	0.011	0.009	0.011	0.005
**0.6**	91.8	0.008	0.011	0.010	0.014	0.011	0.014	0.011	0.014	0.008
**0.8**	92.4	0.010	0.012	0.011	0.017	0.015	0.017	0.014	0.017	0.011
**1.2**	93.6	0.015	0.016	0.015	0.023	0.022	0.022	0.018	0.023	0.016
**1.6**	94.9	0.020	0.020	0.018	0.030	0.029	0.028	0.022	0.029	0.021
**2.0**	96.1	0.025	0.023	0.021	0.036	0.037	0.034	0.026	0.035	0.026
**2.2**	96.7	0.028	0.025	0.023	0.039	0.040	0.037	0.029	0.038	0.029
**2.4**	97.3	0.030	0.027	0.024	0.042	0.044	0.040	0.031	0.041	0.032
**2.6**	97.9	0.033	0.029	0.026	0.045	0.048	0.042	0.033	0.044	0.034
**3.0**	99.1	0.038	0.033	0.029	0.051	0.055	0.048	0.037	0.050	0.040
**3.3**	100.1	0.043	0.036	0.032	0.057	0.062	0.054	0.041	0.056	0.045
**3.6**	101.0	0.045	0.038	0.034	0.060	0.066	0.057	0.044	0.059	0.047
**3.8**	101.6	0.048	0.040	0.035	0.063	0.070	0.060	0.046	0.062	0.050
**4.0**	102.2	0.050	0.042	0.037	0.066	0.074	0.063	0.048	0.065	0.053
**4.2**	102.8	0.053	0.044	0.038	0.069	0.077	0.065	0.050	0.068	0.055
**4.4**	103.4	0.055	0.045	0.040	0.072	0.081	0.068	0.052	0.071	0.058
**4.8**	104.7	0.060	0.049	0.043	0.079	0.088	0.074	0.056	0.077	0.063
**5.0**	105.3	0.063	0.051	0.045	0.082	0.092	0.077	0.059	0.080	0.066

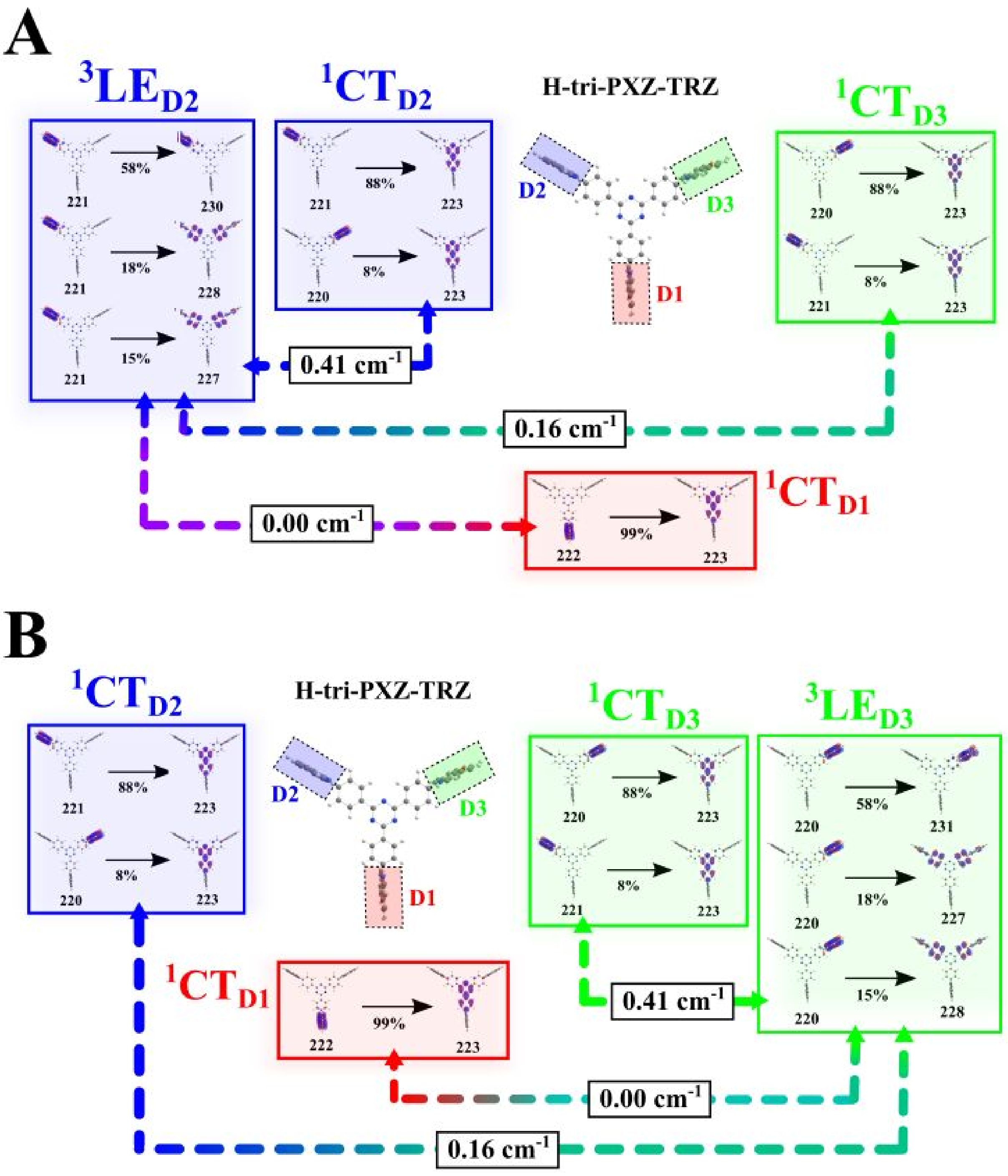

Figure S12. The molecular orbitals involved in electronic transitions between $^{3}LE_{D2}$ (**A**), $^{3}LE_{D3}$ (**B**), and respective ^{1}CT states in **H-tri-PXZ-TRZ**.

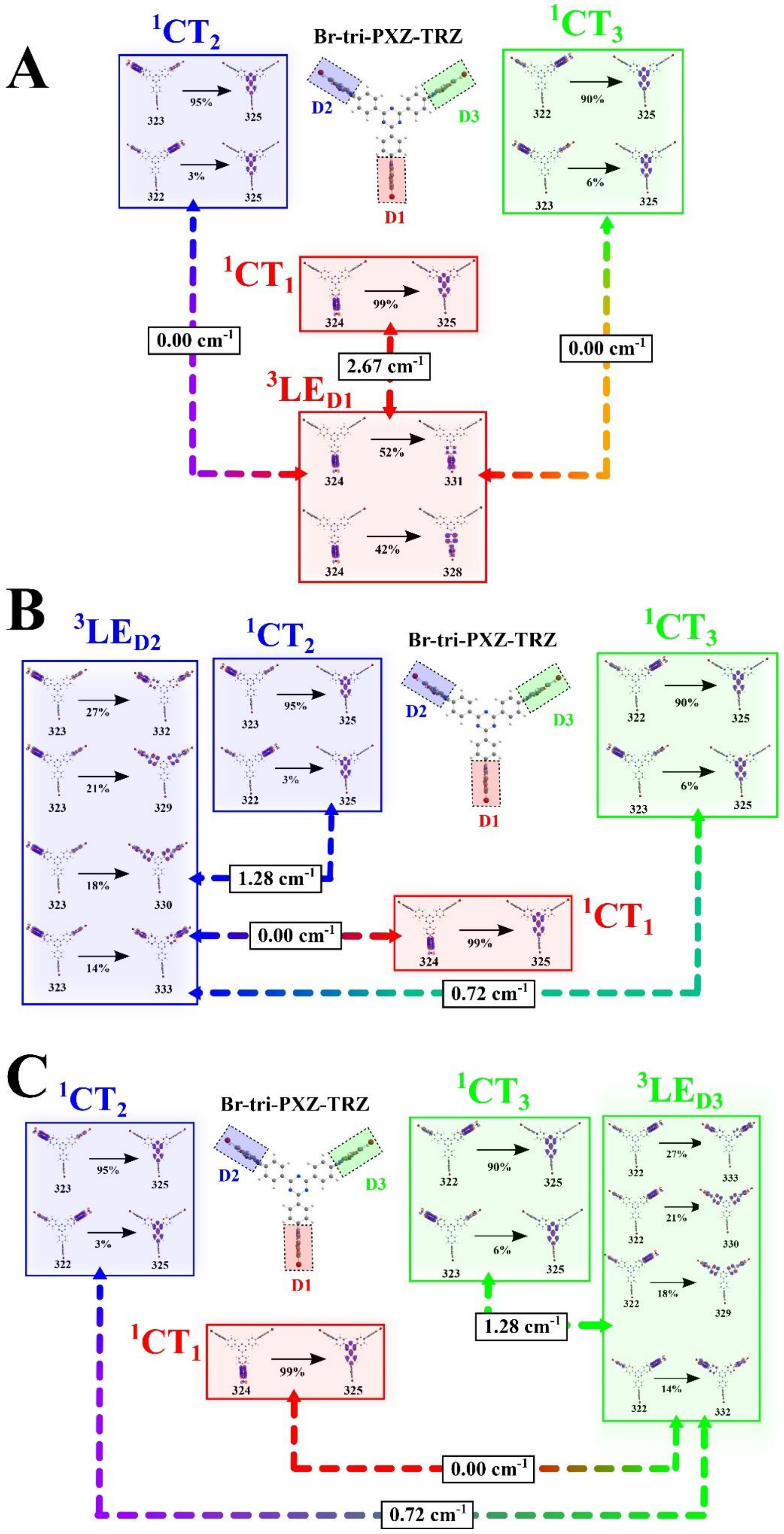

Figure S13. The molecular orbitals involved in electronic transitions between $^3LE_{D1}$ (**A**), $^3LE_{D2}$ (**B**), $^3LE_{D3}$ (**C**) and respective ^1CT states in **Br-tri-PXZ-TRZ**.

Section S4. NMR and MALDI-TOF of Br-tri-PXZ-TRZ

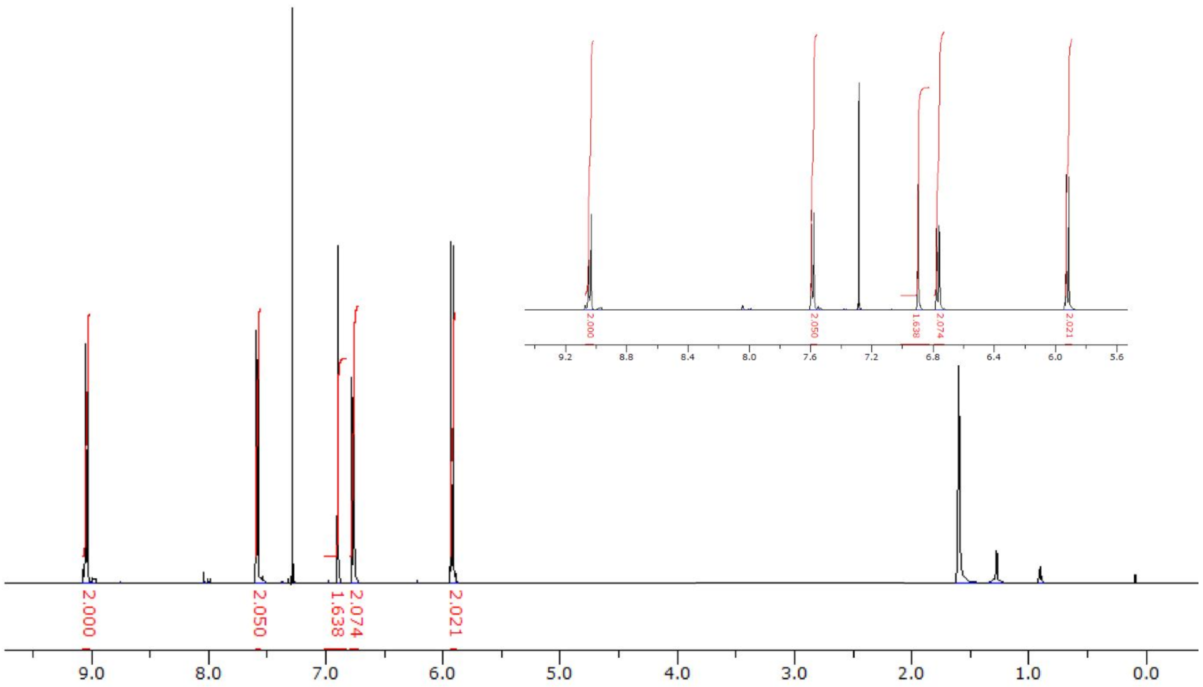

Figure S14. ^{1}H-NMR spectrum of **Br-tri-PXZ-TRZ** in $CDCl_3$

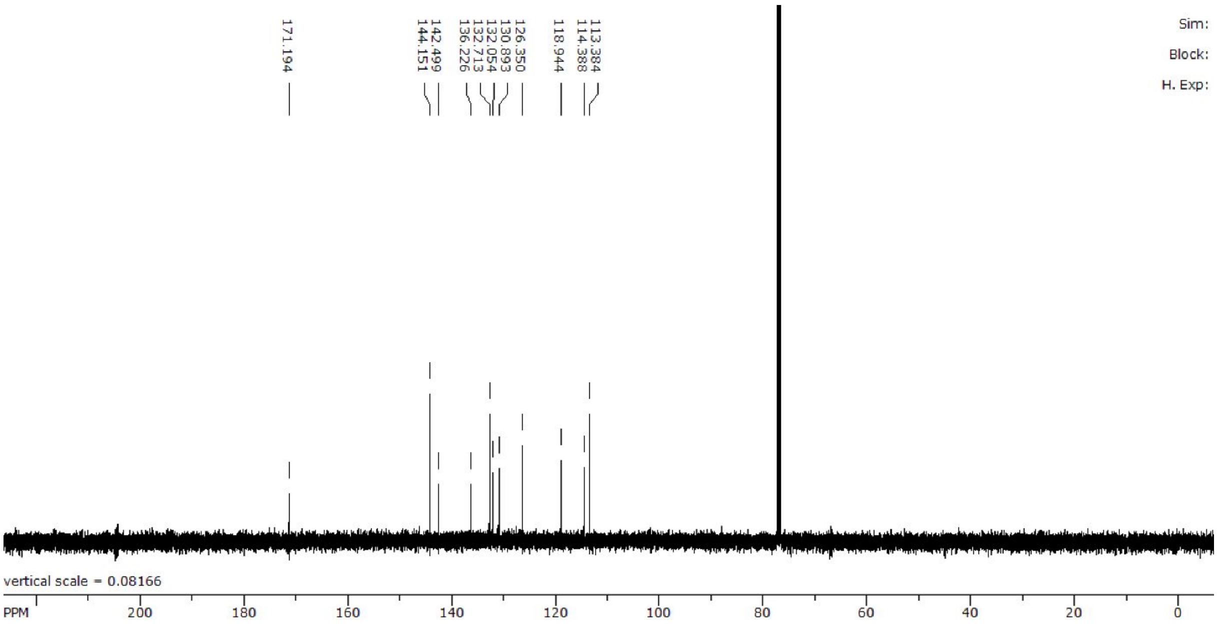

Figure S15. ^{13}C-NMR spectrum of **Br-tri-PXZ-TRZ** in $CDCl_3$

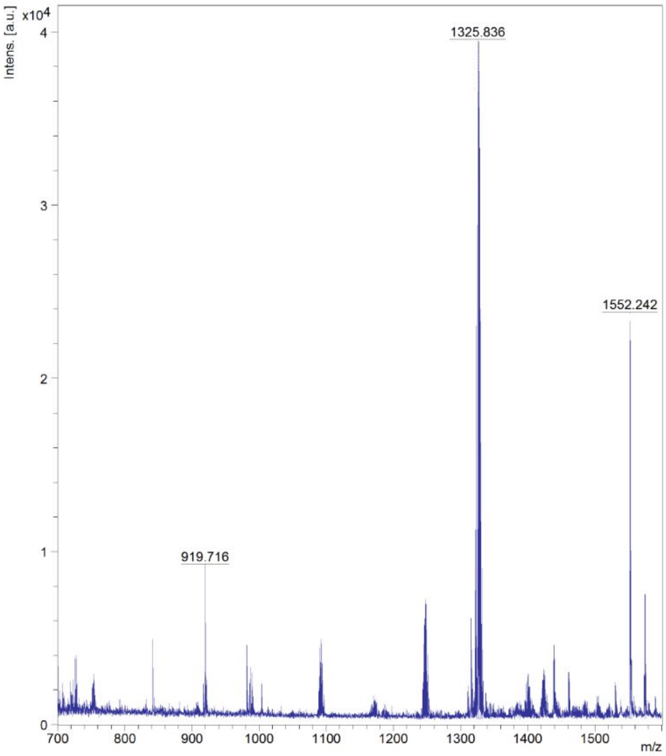

Figure S16. MALDI-TOF spectrum of **Br-tri-PXZ-TRZ** in DHB matrix

References

[1] John R. Taylor *"An Introduction to Error Analysis: The Study of Uncertainties in Physical Measurements"*, Third Edition University of Colorado, **2022**.

[2] Tao, Y.; Yuan, K.; Chen, T.; Xu, P.; Li, H.; Chen, R.; Zheng, C.; Zhang, L.; Huang, W. Thermally Activated Delayed Fluorescence Materials Towards The Breakthrough Of Organoelectronics. Adv. Mater. 2015, 26, 7931 – 7958.

[3] Mońka, M.; Serdiuk, I. E.; Kozakiewicz, K.; Hoffman, E.; Szumilas, J.; Kubicki, A.; Park, S. Y.; Bojarski, P. Understanding the internal heavy-atom effect on thermally activated delayed fluorescence: application of Arrhenius and Marcus theories for spin–orbit coupling analysis. *J. Mater. Chem. C* **2022**, *10*, 7925-7934.

[4] Serdiuk, I. E.; Mońka, M.; Kozakiewicz, K.; Liberek, B.; Bojarski, P.; Park, S. Y. Vibrationally Assisted Direct Intersystem Crossing between the Same Charge-Transfer States for Thermally Activated Delayed Fluorescence: Analysis by Marcus–Hush Theory Including Reorganization Energy. J. Phys. Chem. B 2021, 125, 2696–2706.
